# RNA-Binding Proteins MCPIP3 and IGF2BP3 Antagonize Lung Cancer Metastasis by Reversibly Regulating METAP2 Expression via mRNA Stability

**DOI:** 10.3390/cancers18152384

**Published:** 2026-07-24

**Authors:** Shaoyu Song, Hongwei Li, Ailing Li, Bingwei Li, Xueting Liu, Wenbao Lu, Ruijuan Xiu

**Affiliations:** Institute of Microcirculation, Peking Union Medical College, Chinese Academy of Medical Sciences, Beijing 100005, China; songshaoyu7739@163.com (S.S.); lihw66@imc.pumc.edu.cn (H.L.); lialwork@imc.pumc.edu.cn (A.L.); drli715@163.com (B.L.); 18519231305@163.com (X.L.); xiurj@imc.pumc.edu.cn (R.X.)

**Keywords:** MCPIP3, IGF2BP3, LUAD, metastasis, METAP2

## Abstract

**Background/Objectives**: The homeostatic imbalance between tumor metastasis-promoting genes and metastasis-suppressing genes determines the metastatic potential of lung adenocarcinoma (LUAD) cells. However, the post-transcriptional regulation mechanism mediated by RNA-binding proteins (RBPs) in maintaining the expression balance of tumor metastasis-related genes remains unclear. **Methods**: The expression of MCPIP3 and IGF2BP3 in human lung cancer tissues was analyzed using bioinformatics methods and validated using qRT-PCR, immunohistochemistry, and immunoblotting. In vitro cellular experiments and in vivo animal examinations were used to evaluate the effects of MCPIP3 and IGF2BP3 on metastasis. RNA sequencing, PCRarray, RNA pull-down and mass spectrometry, RNA-EMSA, RNA immunoprecipitation (RIP), mRNA stability, and luciferase assays were performed to elucidate the mechanism. **Results**: MCPIP3 is expressed at low levels, while IGF2BP3 is highly expressed in LUAD tissues. Lower MCPIP3 expression and higher IGF2BP3 expression in lung cancer tissues were significantly associated with poor prognosis in LUAD patients. MCPIP3 significantly inhibited the metastasis of lung cancer cells both in vitro and in vivo, whereas IGF2BP3 promoted metastasis. Mechanistically, MCPIP3 specifically bound to the stem–loop structure in the 3′UTR (untranslated region) of the METAP2 transcript and degraded its mRNA via its RNase domain. In contrast, by interacting with the common stem–loop structure, IGF2BP3 could stabilize METAP2 mRNA and promote its expression. Notably, inverse correlations were observed between the expression of MCPIP3 and IGF2BP3 and the expression of METAP2 in LUAD tissues. **Conclusions**: MCPIP3 and IGF2BP3 antagonistically control lung cancer cell metastasis by balancing the expression of metastasis-promoting genes through mediating mRNA stability, thereby providing potential targets for lung cancer treatment.

## Simple Summary

Tumor metastasis is one of the key causes of cancer-related deaths, including lung cancer. However, the mechanism controlling the expression of metastasis-related genes has still not been completely clarified, especially the post-transcriptional regulation mechanism mediated by RNA-binding proteins (RBPs) in human lung cancer cells. Here, we revealed that the RBPs MCPIP3 (monocyte chemotactic protein-induced protein 3) and IGF2BP3 (insulin-like growth factor 2 mRNA-binding protein 3) negatively and positively regulate lung cancer cell metastasis, respectively, by antagonistically regulating METAP2 (methionine aminopeptidase 2) mRNA stability and expression through targeting the same stem–loop structure. These findings provide new insights into the yin–yang effects of RBPs in mediating metastasis-related gene expression in human lung cancer cells and may provide potential molecular targets for lung cancer treatment.

## 1. Introduction

Lung cancer is among the most common cancers worldwide. Although the 5-year survival rate for lung cancer patients has significantly improved because of surgical treatments and early diagnosis, many patients still experience cancer metastasis, which subsequently leads to organ failure and cancer-related death [1]. Therefore, further elucidation of the mechanisms underlying lung cancer cell metastasis is necessary and may provide promising therapeutic targets. The metastasis of lung cancer cells is determined by the expression of metastasis-promoting genes, such as *Methionine aminopeptidase 2* (*METAP2*) [2], *Harvey rat sarcoma viral oncogene homolog* (*HRAS*) [3], and *Pinin* (*PNN*) [4], and metastasis-inhibiting genes, such as *metastasis suppressor 1* (*MTSS1*) [5], *catenin alpha 1* (*CTNNA1*) [6], and *cadherin 11* (*CDH11*) [7], in cancer cells. Lung cancer cell metastasis is usually activated either by the upregulation of metastasis-promoting genes or by the downregulation of metastasis-suppressing genes. However, the molecular mechanism controlling the expression of metastasis-related genes remains unclear, especially the mechanisms by which multiple RBPs antagonistically regulate the expression of these genes at the post-transcriptional level.

Monocyte chemotactic protein-induced protein 3 (MCPIP3), encoded by the *zc3h12c* gene, is a CCCH-type zinc finger RNA-binding protein belonging to the ZC3H12 family [8]. MCPIP3 has endonuclease activity and degrades mRNA through its RNase domain [9]. Extensive research has focused on the immunomodulatory function of MCPIP3, which can prevent autoimmune reactions by controlling the expression of a pro-inflammatory cytokine gene [10]. MCPIP3-knockout mice develop hypertrophic lymph nodes [11]. MCPIP3 is also involved in the pathogenesis of psoriasis [12] and skin inflammation [13] through the mediation of inflammatory responses. Therefore, MCPIP3 is believed to be an important regulator of inflammation homeostasis. To date, the role of MCPIP3 in human cancer progression remains unclear. It has been reported that the expression of MCPIP3 is closely correlated with the survival of patients with lung cancer [14], colon cancer [15], and kidney cancer [16]. Moreover, MCPIP3 is also involved in regulating the metastasis of human colon cancer cells [17] and is considered a tumor suppressor in colon cancer [18]. However, the role and mechanism of MCPIP3 in human lung cancer development, especially in metastasis progression, remain unclear.

Methionine aminopeptidase 2 (MetAP2), a metallopeptidase known to play an important role in tumor progression, is considered a key promoter of tumor metastasis [19], growth [20], and angiogenesis [21]. METAP2 may be an important functional protein for the treatment and outcome of prostate cancer patients [22]. METAP2 has been found to promote the metastasis of various human cancers, including colon cancer, breast cancer, and ovarian cancer, indicating that METAP2 is an important regulator of cancer metastasis progression [2,23,24]. METAP2 is also believed to be a promising molecular target for treating cancer metastasis [25,26]. However, the mechanism controlling METAP2 gene expression in cells, especially in lung cancer cells, remains unclear.

In this study, we discovered that MCPIP3 acts as a potent lung cancer metastasis suppressor by suppressing the expression of the tumor metastasis-promoting gene *METAP2*. Computational analysis revealed that MCPIP3 expression was significantly reduced among the 1756 RBPs analyzed in lung cancer tissues. Low levels of MCPIP3 in lung cancer tissues were strongly correlated with poor prognosis in lung cancer patients. We found that MCPIP3 was able to inhibit the migration and invasion of lung cancer cells both in vitro and in vivo. MCPIP3 could specifically downregulate the expression of the tumor metastasis-promoting gene *METAP2* by degrading its mRNA through direct binding to the stem–loop structure in the 3′UTR. Interestingly, IGF2BP3, another typical RBP [27], was identified to bind to the stem–loop structure of METAP2 mRNA while stabilizing its mRNA. We further found that IGF2BP3 could antagonize MCPIP3-mediated inhibition of METAP2 expression and promote lung cancer cell metastasis. Moreover, we found that the expression of MCPIP3 and IGF2BP3 was significantly negatively and positively correlated with METAP2 expression, respectively, in human lung cancer tissues. Collectively, these results revealed a post-transcriptional regulatory mechanism of metastasis-promoting genes mediated by the RBPs MCPIP3 and IGF2BP3, which antagonistically control the stability and expression of these genes in lung cancer cells, thereby providing potential molecular targets for lung cancer treatment.

## 2. Materials and Methods

### 2.1. Cell Lines and Plasmids

Human lung cancer cell lines (A549 and NCI-H23) were purchased from the American Type Culture Collection (ATCC) and cultured in PRM1640 medium supplemented with 10% fetal bovine serum (FBS) and 1% penicillin/streptomycin, respectively. HEK293 and HEK293T cells were purchased from the National Infrastructure of Cell Line Resource (Beijing, China) and cultured in Dulbecco’s Modified Eagle Medium supplemented with 10% FBS and 1% penicillin/streptomycin. pEGFP-N1 vectors expressing human *MCPIP3* and *IGF2BP3* were generated by inserting the full-length *MCPIP3* (NM_033390.2) and *IGF2BP3* (NM_006547.3) coding sequences at NheI/SacII and NheI/SacI sites, respectively. The MCPIP3 truncation constructs were generated by subcloning the amplified fragments into pEGFP-N1 vectors at NgeI and SacII sites, respectively. For luciferase reporters, the full-length 3′UTRs of human *MERAP2*, *HRAS*, *PNN*, and *CTBP1* were subcloned into the pGL3 control vector between Xba I and Fse I sites, respectively. To generate the stem–loop mutation reporters, point-mutated and truncated *METAP2*-3′UTR (∆stem–loop) were amplified, sequenced, and subcloned into a pGL3 control vector using a Phusion Site-Directed Mutagenesis Kit (Thermo Scientific, Arbor, MI, USA).

### 2.2. Wound Healing Assay

Lung cancer cells overexpressing or knocking down MCPIP3 were seeded in 35 mm dishes and incubated at 37 °C. After the cells grew into a monolayer, cell scratches were created using 200 μL pipette tips. The cells continued to be cultured for 24–48 h. The images of the scratch confluence were acquired using a microscope (Zeiss, Oberkochen, Germany). The Zeiss ZEN software (ZEN 2 (blue edition)) was used to measure the open area of the wound.

### 2.3. Transwell Invasion Assay

The transwell inserts of 24-well transwell plates (8.0 μm pore, Corning, Corning, NY, USA) were precoated with 50 μL Matrigel (BD Biosciences, San Jose, CA, USA) and incubated for 1 h at 37 °C. Then, MCPIP3-overexpressing lung cancer cells suspended in 200 μL of serum-free medium were seeded in transwell inserts, whereas the lower wells were filled with 800 µL of conditioned medium. After 24 h of culture, the inserts were fixed with 4% paraformaldehyde and stained with 0.1% crystal violet. The remaining cells were removed using a cotton swab from the inner side of the inserts. Each inverted insert was photographed to visualize the invading cells. The number of invaded cells was normalized to the control.

### 2.4. RNA Extraction and Real-Time Quantitative PCR (RT-qPCR)

Total RNA was extracted using TRIzol (Thermo Fisher, Waltham, MA, USA), and genomic DNA was removed with RNase-free DNase. Then, the RNA was reverse-transcribed into cDNA for real-time qPCR using the SYBR green Fast Master Mix (Roche, Basel, Switzerland). The gene expression level was calculated using the ∆∆Ct method and normalized using *GAPDH*. The sequences of primers are shown in Table S1.

### 2.5. PCR Array Assay

Total RNA was collected from MCPIP3-overexpressing A549 cells and empty vector control cells, and then reverse-transcribed into cDNA. The mRNA expression of each metastasis-related gene was measured using the QIAGEN Human Tumor Metastasis PCR Array Kit (Qiagen, Hilden, Germany) (Cat. No. 330231, PAHS-028ZA). Gene expression fold changes were quantified using the 2^–∆∆Ct^ method. Detailed PCRarray data are listed in Table S2.

### 2.6. RNA Sequencing Analysis

Cells from MCPIP3 overexpression groups, IGF2BP3 overexpression groups, and their control groups were collected for RNA sequencing. Total RNA was extracted and enriched using oligo (dT) beads and synthesized into cDNA. RNA sequencing was completed by Allwegene Technology Inc., Beijing, China. The sequencing was performed using Illumina’s second-generation high-throughput sequencing platform and PE150 sequencing strategy. To complete the alignment of transcriptomes, reads of inferior quality or adapters were filtered and further processed using Tophat2 and Cufflinks. Differentially expressed genes and transcripts were identified with a threshold of false discovery rate (FDR)-adjusted *p* ≤ 0.05. The regulated genes are listed in Table S3.

### 2.7. mRNA Stability Assay

Actinomycin D (ActD, 5 µg/mL) and 5,6-dichlorobenzimidazole nucleoside (DRB, 5 µg/mL) were added to the cells to block new RNA synthesis. Relative mRNA levels at different time points were measured using RT-qPCR. mRNA half-lives were determined by comparing the mRNA levels before treatment with ActD and DRB.

### 2.8. RNA-EMSA (Electrophoretic Mobility Shift Assay)

The probes labeled with 3′-end biotin were incubated with whole-cell lysates containing the MCPIP3/GFP fusion protein in binding buffer at room temperature for 30 min. RNA–protein complexes (RPCs) were separated using electrophoresis with a non-denaturing polyacrylamide gel (6%). The RPCs were then transferred onto a nylon membrane and crosslinked by UV irradiation. According to the manufacturer’s guide, the membrane was detected using the LightShift chemiluminescent EMSA kit (Pierce).

### 2.9. Western Blot Analysis

Cells or tissue samples were lysed with RIPA buffer containing PMSF and a protease inhibitor cocktail (Roche). Protein concentration was determined using a BCA assay. Equal protein lysates were separated on SDS–PAGE gels and then transferred onto PVDF membranes using Bio-Rad electrotransfer (Bio-Rad, Hercules, CA, USA). The membranes were then blocked in 5% non-fat milk for 1 h and incubated overnight at 4 °C with the primary antibody (1:1000 in 5% fat-free milk). After being washed three times with TBST, membranes were incubated with HRP-conjugated secondary antibodies (1:5000 in 5% fat-free milk) for 1 h at room temperature. Protein bands were detected using an ECL chemiluminescent detection kit (#34077, Pierce; Rockford, IL, USA). The antibodies are listed in File S1. Uncropped Western blot images are shown in File S2.

### 2.10. RNA Pull-Down and Mass SPECTROMETRY

3′-end biotin-labeled RNA probes with or without stem–loop structures were used for RNA pull-down. Whole-cell lysates were obtained from MCPIP3-overexpressing A549 cells and were precleared with streptavidin-coated Dynabeads (M-280, Invitrogen, Carlsbad, CA, USA). The precleared lysates were incubated with RNA probes and Dynabeads for 2 h at 4 °C. The beads were then washed six times and boiled in SDS–PAGE sample buffer. The coprecipitated proteins were separated by SDS–PAGE gel, and then the gel underwent silver staining. The unique bands were excised and in-gel digested with trypsin for mass spectrometry (LC–MS) analysis. Mass spectrometry and analysis were conducted by Beijing Qinglian Biotech Co., Ltd. (Beijing, China) using the RIGOL L-3000 HPLC System (RIGOL, Beijing, China). The identified proteins are listed in Table S4.

### 2.11. Animal Study

For this, 6–8-week-old female BALB/c nude mice were purchased from the Institute of Laboratory Animal Science, Chinese Academy of Medical Sciences (CAMS) & Peking Union Medical College (PUMC) and maintained under specific pathogen-free conditions. A549/MCPIP3-GFP (2.0 × 10^6^/100 µL PBS), A549/GFP cells (2.0 × 10^6^/100 µL PBS), A549/shMCPIP3 (2.0 × 10^6^/100 µL PBS), A549/Scramble cells (2.0 × 10^6^/100 µL PBS), and A549/IGF2BP3-GFP (2.0 × 10^6^/100 µL PBS) were subcutaneously injected into the right flank of nude mice. Tumor volumes were measured every other day and calculated using the formula: length × width × height (mm^3^). At the end of the experiments, mice were sacrificed, and the tumors and whole lung tissues were harvested. The experimental procedures were approved by the Experimental Animal Care and Ethics Committee of the Institute of Microcirculation, CAMS & PUMC.

### 2.12. Statistical Analysis

Statistical analysis between groups was performed using GraphPad Prism 9. Data were expressed as the mean ± SD based on at least three independent repeats. The statistical analyses used Student’s *t*-tests for comparisons between the treatment and the vehicle control. Spearman’s correlation analysis was used to evaluate the relationship between two different gene expression profiles. Statistical significance was considered when *p* < 0.05.

## 3. Results

### 3.1. MCPIP3 Expression Is Reduced in Human Lung Cancer Tissues and Is Associated with Poor Survival

**Figure 1:**
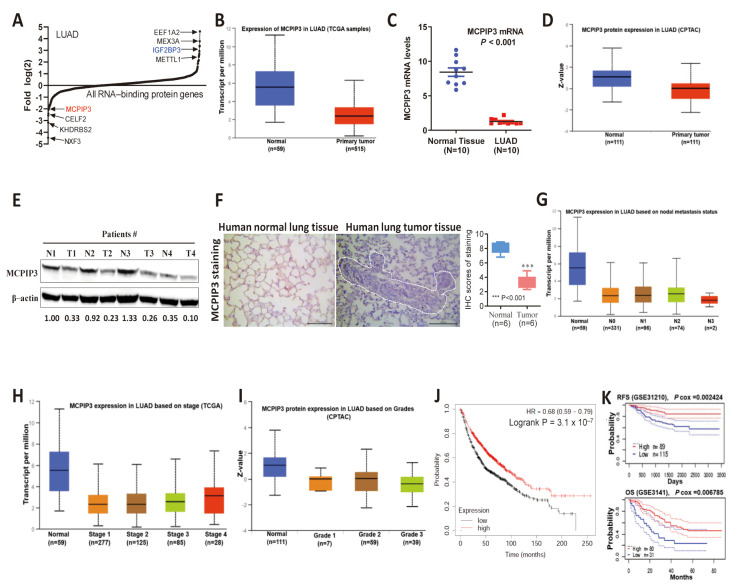
MCPIP3 expression is reduced in human lung cancer tissues and is associated with poor survival. (**A**) The expression of 1756 RNA-binding protein genes in human LUAD was profiled based on the TCGA database. (**B**) Expression of MCPIP3 in human LUAD and normal tissues was analyzed using the UALCAN online tool (https://ualcan.path.uab.edu/analysis-prot.html (accessed on 8 May 2026)). (**C**) MCPIP3 expression was tested using qRT-PCR in human LUAD specimens (*n* = 10) and their surrounding “normal” tissues (*n* = 10). (**D**) MCPIP3 protein expression in human LUAD and normal tissues was shown based on the CPTAC database (http://ualcan.path.uab.edu/analysis-prot.html (accessed on 8 May 2026)). (**E**) MCPIP3 protein expression in several LUAD tissues and their paired normal tissues was displayed using Western blots. (**F**) Representative images of IHC staining for MCPIP3 in human normal lung tissues and LUAD tissues (left). Scale bar, 100 μm. MCPIP3 IHC staining scores of 6 pairs of matched LUAD and normal tissues (right). (**G**) MCPIP3 transcript expression levels were reduced with the nodal metastasis status in LUAD patients (http://ualcan.path.uab.edu/analysis-prot.html (accessed on 8 May 2026)). (**H**,**I**) MCPIP3 transcript (**H**) and protein (**I**) expression were analyzed by the main pathological stages of LUAD. (**J**) Kaplan–Meier overall survival curves of lung cancer patients (*n* = 2167) grouped according to MCPIP3 expression levels (https://kmplot.com/analysis/ (accessed on 8 May 2026)). (**K**) Kaplan–Meier relapse-free survival (RFS) and overall survival (OS) curves of LUAD patients with low and high tumor MCPIP3 expression in the GSE31210 and GSE3141 datasets, respectively.

To elucidate the expression of RBPs in human lung cancer, the mRNA expression of 1756 RBPs was analyzed using The Cancer Genome Atlas (TCGA) datasets. We found that MCPIP3 is lowly expressed in lung adenocarcinoma (LUAD) and lung squamous cell carcinoma (LUSC) tissues and ranked among the lowest candidates (Figure 1A and Figure S1A). The expression patterns of several known RBPs were also identified, such as KH domain-containing, RNA-binding, signal transduction-associated protein 2 (KHDRBS2) [28], CUGBP Elav-like family member 2 (CELF2) [29], IGF2BP3 [30], and tRNA (guanine-N(7))-methyltransferase (METTL1) [31] (Figure 1A), further confirming the reliability of our computational analysis. We further found that the expression of MCPIP3 mRNA was significantly reduced in LUAD (Figure 1B) and LUSC (Figure S1B,C) tissues. MCPIP3 expression was also lower in our collected LUAD tissues than in the adjacent non-cancerous tissues (Figure 1C). MCPIP3 protein expression was also reduced in LUAD tissues (Figure 1D). Western blot and immunohistochemistry (IHC) results also confirmed a low level of MCPIP3 expression in LUAD tissues (Figure 1E,F). Notably, the expression of MCPIP3 decreases with increasing lymph node metastasis in patients with LUAD (Figure 1G) and LUSC (Figure S1D). We further found that MCPIP3 expression was lower in LUAD and LUSC tissues at higher stages (Figure 1H and Figure S1E), and that the protein expression of MCPIP3 decreased with increasing cancer grade in LUAD patients (Figure 1I). These findings strongly suggest that MCPIP3 expression might be closely associated with human lung cancer metastasis. Additionally, the results of Kaplan–Meier analyses indicated that lower MCPIP3 expression in lung cancer tissues was significantly associated with poor survival in lung cancer patients (Figure 1J,K). Overall, these results suggest that MCPIP3 expression is suppressed in LUAD tissues and is strongly associated with lung cancer metastasis.

### 3.2. MCPIP3 Inhibits Lung Cancer Cell Metastasis by Mediating the Expression of Tumor Metastasis-Related Genes

**Figure 2:**
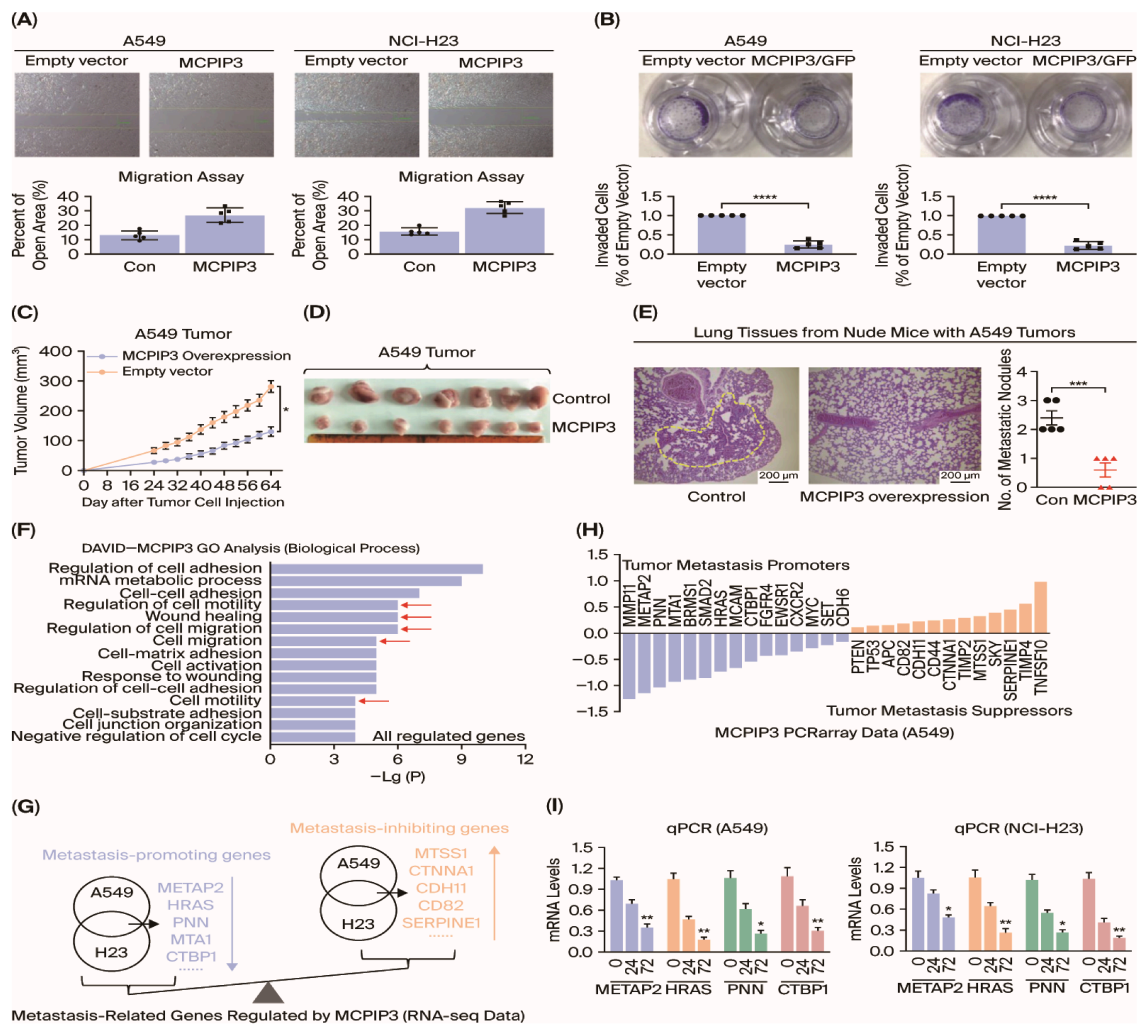
MCPIP3 inhibits lung cancer cell metastasis by mediating the expression of tumor metastasis-related genes. (**A**) Representative images (**top**) and analysis results (**bottom**) of wound healing assays with MCPIP3-overexpressing A549 cells, MCPIP3-overexpressing NCI-H23 cells, and their control groups. (**B**) Representative images (**top**) and analysis (**bottom**) of transwell invasion assays with MCPIP3-overexpressing A549 cells, MCPIP3-overexpressing NCI-H23 cells, and their control groups. (**C**) A549 tumor growth curves in nude mice (*n* = 6) after overexpressing MCPIP3. (**D**) Picture of A549 tumors expressing MCPIP3 or a control vector. (**E**) Representative HE staining images of lung tissues from nude mice bearing A549/MCPIP3 or A549/Control vector tumors. Scale bar, 100 μm. **Right** panel shows the quantification of metastatic nodules. (**F**) GO (Gene Ontology) analysis of all regulated genes by MCPIP3 using the DAVID online tool (https://davidbioinformatics.nih.gov/ (accessed on 9 May 2026)), and several cell-motility-related terms are indicated by red arrows. (**G**) RNA-seq data showed that multiple metastasis-promoting genes were commonly downregulated, while the metastasis-inhibiting genes were commonly upregulated in MCPIP3-overexpressing A549 and NCI-H23 cells. (**H**) PCR Array data showing the metastasis promoters were downregulated, and the metastasis suppressors were upregulated in MCPIP3-overexpressing A549 cells. (**I**) qPCR data showing the mRNA expression levels of metastasis-promoting genes (METAP2, HRAS, PNN, and CTBP1) in MCPIP3-overexpressing A549 and NCI-H23 cells. Data are presented as mean ± SD; * *p* < 0.05, ** *p* < 0.01, *** *p* < 0.001, **** *p* < 0.0001, in an unpaired *t*-test.

Next, we investigated the effect of MCPIP3 on the metastasis of lung cancer cells. Exogenous MCPIP3 was expressed in A549 cells and NCI-H23 cells, respectively (Figure S2A). Ectopic expression of MCPIP3 significantly inhibited the migration (Figure 2A) and invasion (Figure 2B) of lung cancer cells in vitro. In vivo experimental results further revealed that, compared with the control treatment, the overexpression of MCPIP3 significantly suppressed the growth (Figure 2C,D) and lung metastasis of A549 tumors (Figure 2E), indicating that MCPIP3 could effectively inhibit lung cancer cell metastasis. Next, RNA sequencing (RNA-seq) was conducted to identify the downstream genes regulated by MCPIP3 in lung cancer cells. A total of 4971 genes were commonly upregulated, while 5290 genes were commonly downregulated by MCPIP3 (Figure S2B; Table S3). Gene Ontology (GO) analysis revealed that several terms associated with cancer cell metastasis, including cell migration, wound healing, and cell motility (Figure 2F), were significantly enriched, indicating that MCPIP3 might regulate the expression of tumor metastasis-related genes in lung cancer cells. We found that MCPIP3 could downregulate the expression of a series of metastasis-promoting genes (METAP2, HRAS, PNN, and CTBP1) and upregulate the expression of many metastasis-suppressing genes (MTSS1, CTNNA1, and CDH11) in A549 and NCI-H23 cells (Figure 2G). To further identify the genes affected by MCPIP3 in the tumor metastasis signaling pathway, PCR array detection was performed using the Human Tumor Metastasis RT^2^ Profiler PCR Array (QIAGEN). Indeed, we found that a total of 15 metastasis-promoting genes were downregulated, and 13 metastasis-suppressing genes were upregulated in A549 cells overexpressing MCPIP3 (Figure 2H). Although the upregulated and downregulated genes were not exactly the same due to differences in the sensitivities of detection methods, the trend of MCPIP3 downregulating the expression of metastasis-promoting genes and upregulating the expression of metastasis-suppressing genes was very clear. In addition, qPCR results confirmed that the mRNA expression of four metastasis-promoting genes (METAP2, HRAS, PNN, and CTBP1) was downregulated, whereas the expression of four metastasis-suppressing genes (MTSS1, CTNNA1, CDH11, and CD82) was upregulated in lung cancer cells overexpressing MCPIP3 (Figure 2I and Figure S2C), further validating our RNA-seq and PCR array results. Taken together, these data demonstrate that MCPIP3 can inhibit the metastasis of human lung cancer cells by regulating the expression of tumor metastasis-related genes.

### 3.3. MCPIP3 Selectively Downregulated the Expression of the Metastasis-Promoting Gene METAP2 by Destabilizing Its mRNA via the RNase Domain

**Figure 3:**
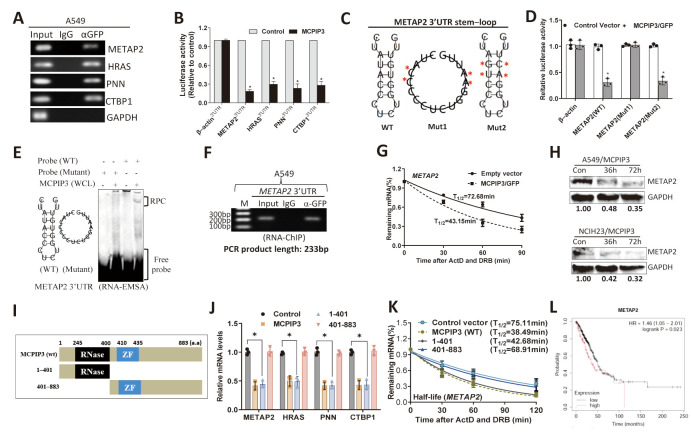
MCPIP3 selectively downregulated the expression of the metastasis-promoting gene METAP2 by destabilizing its mRNA via the RNase domain. (**A**) RNA-IP with specific anti-GFP antibody or isotype IgG, followed by RT-PCR detection, showed that the metastasis-suppressing genes were precipitated by MCPIP3. (**B**) Luciferase reporter assay analysis showing the effects of MCPIP3 overexpression on the luciferase activities of the indicated reporters. (**C**) Mutation strategy of the METAP2 stem–loop structure (asterisks indicate base substitution). The stem–loop structure was deleted in Mut1 (**middle**), while Mut2 retained the stem–loop structure (**right**). (**D**) The relative luciferase activities of the indicated reporters were determined using a reporter assay. (**E**) RNA-EMSA showing the unique RNA–protein complex (RPC) formed using METAP2 stem–loop probes and whole-cell lysates (WCL) extracted from A549/MCPIP3-GFP cells. (**F**) The RNA-ChIP assay was performed with A549/MCPIP3-GFP cells. (**G**) Half-life of the METAP2 mRNA was reduced by MCPIP3 in A549 cells. (**H**) METAP2 and GAPDH protein expression levels were measured using Western blotting in MCPIP3-overexpressing A549 and NCI-H23 cells, respectively. (**I**) Schematic representation of MCPIP3 truncation mutants and their domains. ZF: zinc finger. (**J**) The mRNA expression of four metastasis-promoting genes was measured by qPCR after overexpressing the truncations in A549 cells. (**K**) Half-lives of METAP2 mRNA were measured after overexpression of wild-type (WT) MCPIP3 or its mutants in A549 cells. (**L**) Kaplan–Meier overall survival curves of LUAD patients grouped based on METAP2 mRNA expression (https://kmplot.com/analysis (accessed on 8 May 2026)). Data are presented as mean ± SD; * *p* < 0.05, in unpaired *t*-tests.

Next, we aimed to investigate how MCPIP3 regulates the expression of metastasis-related genes in lung cancer cells. Given that MCPIP3 is considered an RBP, we investigated whether it directly binds to the transcripts of metastasis-related genes in lung cancer cells. RNA immunoprecipitation (RIP) was performed using specific anti-GFP antibodies and isotype IgG, followed by RT-PCR detection. We found that the metastasis-promoting genes, but not those of metastasis-suppressing genes, could be amplified in the complexes precipitated by the anti-GFP antibody (Figure 3A and Figure S3A), indicating that MCPIP3 specifically binds to metastasis-promoting genes but not metastasis-suppressing genes. Since MCPIP family proteins recognize the 3′UTRs of their target genes, we further investigated whether MCPIP3 binds to the 3′UTRs of metastasis-promoting genes. A luciferase reporter assay revealed that MCPIP3 downregulated the activity of a luciferase reporter containing the 3′UTRs of metastasis-promoting genes (Figure 3B and Figure S3B), indicating that MCPIP3 binds to these mRNAs by targeting the 3′UTR. Previous studies have shown that MCPIPs can interact with the stem–loop structure in the 3′UTR [32]. We found that similar stem–loop structures exist in the 3′UTRs of metastasis-promoting genes by aligning the 3′UTR sequences of different species (Figure S3C). To verify whether MCPIP3 also targets the stem–loop structure of metastasis-promoting genes, two METAP2 3′UTR mutant reporters were constructed, in which several nucleotides were substituted; Mut1 lost its stem–loop structure, whereas Mut2 retained it (Figure 3C). Deletion of the stem–loop structure prevented MCPIP3 from inhibiting luciferase activity (Figure 3D). However, restoration of the stem–loop structure enabled MCPIP3 to suppress reporter activity, suggesting that the stem–loop structure is crucial in allowing MCPIP3 to bind to the 3′UTR (Figure 3D). RNA-EMSA results also confirmed that only the stem–loop probes, not the mutated linearized probes, could form a specific RNA–protein complex with MCPIP3 (Figure 3E). Moreover, the METAP2 stem–loop sequence was amplified in the complexes precipitated with the anti-GFP antibody, but not in the group with isotype IgG, as shown by the RNA immunoprecipitation–chromatin immunoprecipitation (RIP-ChIP) results (Figure 3F), meaning that MCPIP3 binds to the 3′UTR of METAP2 mRNA in vivo. We further found that MCPIP3 could effectively reduce the half-lives of METAP2, PNN, and CTBP1 mRNAs in lung cancer cells (Figure 3G and Figure S3D), which was consistent with previous reports [33]. Western blot analysis also confirmed that METAP2 protein expression was downregulated in a time-dependent manner in MCPIP3-overexpressing lung cancer cells (Figure 3H).

MCPIP proteins have been reported to degrade mRNA through the RNase domain. To determine whether the RNase domain is also responsible for MCPIP3-mediated mRNA degradation, two truncation mutants were generated: the amino acid 1–401 (aa 1–401) mutant, which contains the RNase domain, and the aa 401–883 mutant, which lacks the RNase domain (Figure 3I). We found that wild-type (wt) and aa 1–401 mutant MCPIP3 could effectively inhibit the expression and reduce the half-life of METAP2 mRNA, while the aa 401–883 mutant MCPIP3 completely lost this inhibitory effect (Figure 3I–K). These data suggest that the RNase domain is crucial for MCPIP3-mediated metastasis-promoting mRNA degradation. Additionally, we showed that the expression level of METAP2 was significantly negatively correlated with the survival of LUAD patients (Figure 3L). Taken together, these findings indicated that MCPIP3 can effectively reduce the stability and expression of the metastasis-promoting METAP2 gene transcript in human lung cancer cells through its RNase domain.

### 3.4. Knockdown of MCPIP3 Increases the Stability of METAP2 mRNA and Promotes Lung Tumor Metastasis Progression

**Figure 4:**
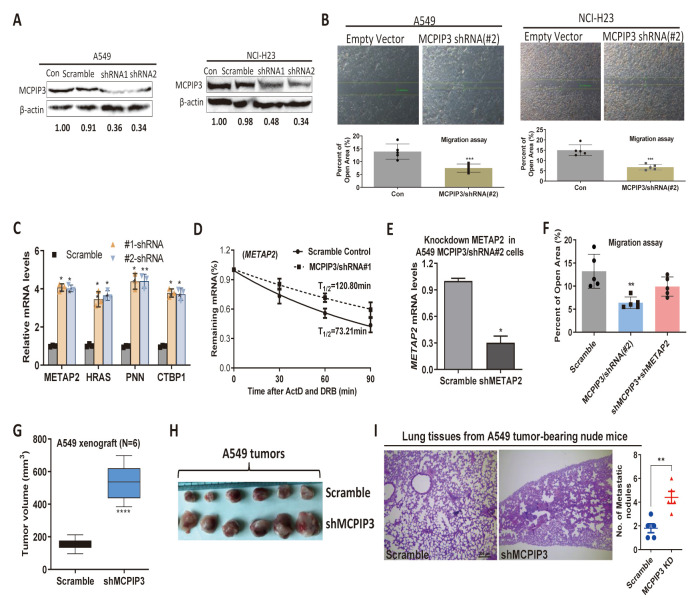
Knockdown of MCPIP3 increases the stability of METAP2 mRNA and promotes lung tumor metastasis progression. (**A**) Knockdown of MCPIP3 protein expression was confirmed by Western blotting in A549 and NCI-H23 cells, respectively. Protein expression quantification was assessed using ImageJ (v1.47). (**B**) Representative images (**top**) and analysis (**bottom**) of lung cancer cell migration after MCPIP3 knockdown. (**C**) mRNA expression of metastasis-promoting genes was measured using qPCR in MCPIP3-silencing A549 cells. (**D**) Half-life of METAP2 mRNA was measured in MCPIP3-knockdown A549 cells. (**E**) mRNA expression of METAP2 mRNA was measured using qPCR in MCPIP3-silencing A549 cells. (**F**) Quantification of cell migration abilities after co-knockdown of MCPIP3 and METAP2 in A549 cells. (**G**) The volumes of MCPIP3-silencing A549 xenograft tumors (*n* = 6). (**H**) Images of MCPIP3-silenced A549 tumors are shown. (**I**) H&E staining of lung tissues from nude mice bearing A549/shMCPIP3 or scramble control tumors. Scale bar, 100 μm. **Right**: quantification of metastatic nodules is shown. Data are presented as mean ± SD; * *p* < 0.05, ** *p* < 0.01, *** *p* < 0.001, **** *p* < 0.0001, in unpaired *t*-tests.

To further confirm the inhibitory effect of MCPIP3 on lung cancer cell metastasis, MCPIP3 was knocked down in human lung cancer cells using two shRNAs. Approximately 54% and 65% of MCPIP3 protein expression was silenced by shRNA#1 and shRNA#2, respectively, in human lung cancer cells (Figure 4A). Suppression of MCPIP3 expression significantly promoted lung cancer cell migration (Figure 4B) and increased the expression of metastasis-promoting genes (Figure 4C). We further found that the half-lives of METAP2 and PNN mRNAs were increased after MCPIP3 knockdown in lung cancer cells (Figure 4D and Figure S4A). To further confirm the involvement of METAP2 in the MCPIP3-mediated inhibition of lung cancer cell metastasis, the METAP2 gene was knocked down by infecting A549 MCPIP3/shRNA#2 cells with an shRNA-expressing lentivirus (Figure 4E). Compared with MCPIP3 knockdown alone, co-knockdown of MCPIP3 and METAP2 partially restored the migration ability of A549 cells to the control group’s level, indicating that the metastasis-promoting gene METAP2 is indeed involved in the MCPIP3-mediated inhibition of lung cancer cell metastasis (Figure 4F and Figure S4B). In vivo experiments further demonstrated that silencing MCPIP3 promoted the growth (Figure 4G,H) and metastasis (Figure 4I) of A549 xenograft tumors. These results demonstrated that reducing MCPIP3 expression increases the expression of metastasis-promoting genes and facilitates lung cancer cell metastasis.

### 3.5. The RNA-Binding Protein IGF2BP3 Interacts with the Common Stem–Loop Structure to Stabilize METAP2 mRNA and Promote Lung Cancer Metastasis

**Figure 5:**
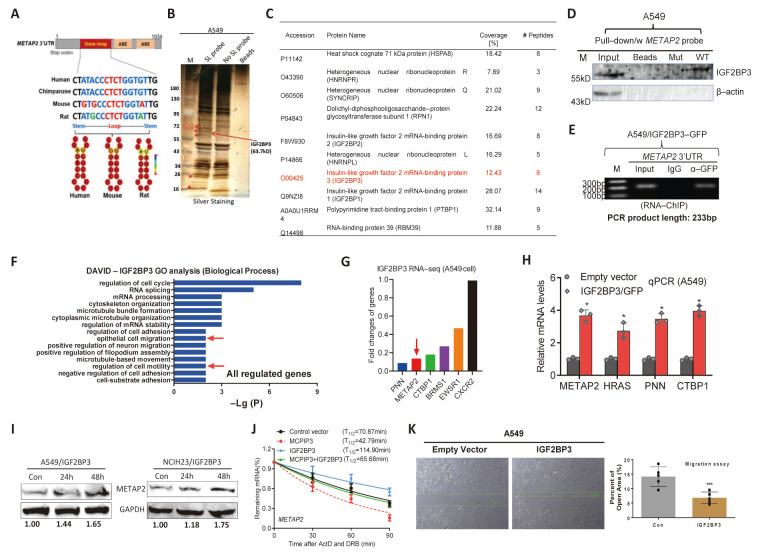
The RNA-binding protein IGF2BP3 interacts with the common stem–loop structure to stabilize METAP2 mRNA and promote lung cancer metastasis. (**A**) Schematic representation of the METAP2 stem–loop structure in the 3′UTRs of different species. (**B**) Silver staining of proteins precipitated by the human METAP2 stem–loop structure in A549 cells. (**C**) Top 10 proteins identified by mass spectrometry are listed. (**D**) RNA pull-down followed by immunoblotting analysis showing that the IGF2BP3 protein was detected in the precipitants pulled down with the METAP2 wild-type (WT) stem–loop probe in A549 cells. (**E**) RIP-ChIP analysis of IGF2BP3 bound to the METAP2 stem–loop sequence in A549/IGF2BP3-GFP cells. (**F**) GO analysis of all regulated genes by IGF2BP3 in A549 cells. The cell-motility-related terms are indicated by red arrows. (**G**) RNA-seq data showing the expression of the indicated metastasis-promoting genes after IGF2BP3 overexpression in A549 cells. (**H**) The mRNA expression of the indicated metastasis-promoting genes was measured using qPCR in IGF2BP3-overexpressing A549 cells. (**I**) Protein expression of METAP2 in IGF2BP3-overexpressing A549 and NCI-H23 cells, as measured by Western blotting. (**J**) Half-life analysis of METAP2 mRNA after MCPIP3 and IGF2BP3 co-overexpression in A549 cells. (**K**) Representative images and analysis of cell migration after IGF2BP3 overexpression in A549 cells. Data are presented as mean ± SD; * *p* < 0.05, *** *p* < 0.001, in unpaired *t*-tests.

Interestingly, although several nucleotide differences exist in the stem–loop sequences of METAP2 among humans, mice, and rats, these conserved sequences can fold into a stem–loop structure (Figure 5A), indicating that this structural stem–loop conformation might be important for controlling METAP2 gene expression. Given that MCPIP3 can destabilize METAP2 mRNA by binding to the stem–loop structure, and METAP2 is highly expressed in human lung cancer cells, it is likely that other RBPs stabilize METAP2 mRNA and increase its expression by targeting the common stem–loop structure. Thus, we performed RNA pull-down experiments, combined with mass spectrometry analysis, to confirm this possibility. For the preparation of linearized RNA probes, the RNA structure was eliminated by heating RNA to 95 °C and then rapidly cooling it on ice. As shown by the silver staining results, many unique protein bands appeared in the group precipitated with stem–loop RNA probes but not in the group with linearized RNA probes (Figure 5B). We also noticed that many known RBPs, including IGF2BP1-3, polypyrimidine tract-binding protein 1 (PTBP1), and RNA-binding motif protein 39 (RBM39), were identified based on their molecular weight and peptide number, further confirming the reliability of our RNA pull-down results (Figure 5C; Table S4). IGF2BP3 was selected to verify its interaction with the METAP2 stem–loop structure. Indeed, IGF2BP3 expression was detected in pulled-down proteins using stem–loop probes but not in the group with linearized probes, as demonstrated by the results of RNA pull-down followed by Western blotting (Figure 5D). Moreover, RIP-CHIP results revealed that the METAP2 stem–loop sequence could be amplified from the RNAs precipitated using the anti-GFP antibody but not from the isotype IgG group (Figure 5E). These results indicate that IGF2BP3 indeed interacted with the stem–loop structure of METAP2 in human lung cancer cells. Next, we examined the genes regulated by IGF2BP3 using RNA-seq in IGF2BP3-overexpressing A549 cells. Interestingly, GO analysis revealed that the genes regulated by IGF2BP3 were significantly enriched in terms related to cell migration (Figure 5F), suggesting that IGF2BP3 may regulate the expression of metastasis-related genes in human lung cancer cells. Notably, several metastasis-promoting genes, including PNN, METAP2, and CTBP1 (Figure 5G,H and Figure S5A), were upregulated by IGF2BP3 (Table S3), whereas their expression was downregulated by MCPIP3. These findings suggested that IGF2BP3 and MCPIP3 regulate the expression of metastasis-promoting genes in human lung cancer cells in a positive and negative manner, respectively. Moreover, METAP2 protein expression was also increased by IGF2BP3 in human lung cancer cells (Figure 5I). Importantly, IGF2BP3 effectively antagonized the MCPIP3-induced shortening of the half-life and reduction in the expression of METAP2 mRNA in human lung cancer cells (Figure 5J and Figure S5B). As expected, we found that IGF2BP3 overexpression significantly promoted A549 cell migration (Figure 5K). Overall, our results demonstrated that IGF2BP3 stabilizes METAP2 mRNA by targeting the stem–loop structure and promoting lung cancer cell migration.

### 3.6. IGF2BP3 and MCPIP3 Potentially Antagonize Lung Cancer Cell Metastasis by Reversibly Modulating METAP2 Expression

**Figure 6:**
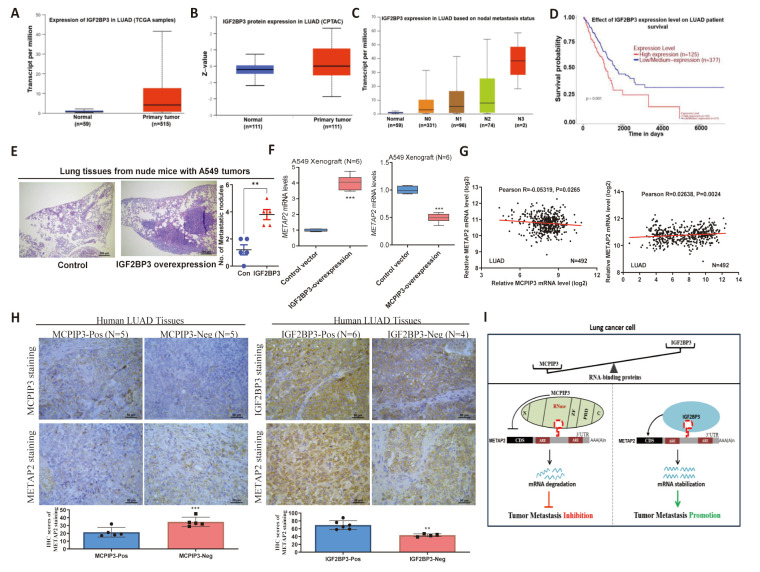
IGF2BP3 and MCPIP3 potentially antagonize lung cancer cell metastasis by reversibly modulating METAP2 expression. (**A**) mRNA expression of IGF2BP3 in LUAD and normal lung tissues was analyzed using the UALCAN online tool. (**B**) Protein expression of IGF2BP3 in human LUAD and normal tissues was displayed by UALCAN. (**C**) IGF2BP3 mRNA expression was increased with the nodal metastasis status in LUAD patients. (**D**) Kaplan–Meier overall survival curves of LUAD patients grouped based on IGF2BP3 mRNA expression level (https://ualcan.path.uab.edu/index.html (accessed on 10 May 2026)). (**E**) H&E staining of lung tissues from nude mice bearing A549/IGF2BP3 tumors or A549/control vector tumors, respectively. Scale bar, 200 μm. Quantification of metastatic nodules is shown in the **right** panel. (**F**) The mRNA expression of METAP2 was measured by qRT-PCR in IGF2BP3-overexpressing and MCPIP3-overexpressing xenograft tumors and their controls (*n* = 6), respectively. (**G**) Pearson’s correlation analysis between MCPIP3, IGF2BP3, and METAP2 expression in human LUAD patients (http://www.oncolnc.org/ (accessed on 12 May 2026)). (**H**) Representative images of IHC staining for MCPIP3 (**left**, **top**), IGF2BP3 (**right**, **bottom**), and METAP2 (**lower**) in human LUAD tissues. Scale bars, 50 μm. (**I**) A proposed model showing the underlying mechanism by which MCPIP3 and IGF2BP3 regulate the metastatic potential of lung cancer cells. Data are presented as mean ± SD; ** *p* < 0.01, *** *p* < 0.001, in unpaired *t*-tests.

Next, we analyzed the expression pattern of IGF2BP3 in human lung cancer tissues. Both IGF2BP3 mRNA (Figure 6A and Figure S6A) and protein (Figure 6B and Figure S6B) expression were significantly increased in LUAD and LUSC tissues. Notably, IGF2BP3 expression increased with increasing degrees of lymph node metastasis in LUAD patients (Figure 6C), which was opposite to the trend observed for MCPIP3 expression (Figure 1G). Similarly, high levels of IGF2BP3 expression in cancer tissues were significantly associated with poor survival in LUAD patients (Figure 6D), whereas low levels of MCPIP3 expression were closely associated with poor survival (Figure 1J). Unsurprisingly, IGF2BP3 promoted the growth (Figure S6C) and metastasis (Figure 6E) of the A549 tumor model, which is consistent with the findings of previous reports [34]. We further discovered that METAP2 expression was significantly increased in IGF2BP3-overexpressing xenografts, but decreased in MCPIP3-overexpressing xenografts (Figure 6F). Moreover, METAP2 expression was significantly negatively correlated with MCPIP3 expression but positively correlated with IGF2BP3 expression in LUAD tissues (Figure 6G). Histological analysis also revealed that the staining intensity of METAP2 was significantly greater in MCPIP3-negative (MCPIP3-Neg) LUAD tissues than in MCPIP3-positive (MCPIP3-Pos) LUAD tissues (Figure 6H). However, the staining intensity of the METAP2 protein was higher in IGF2BP3-positive (IGF2BP3-Pos) LUAD tissues than in IGF2BP3-negative (IGF2BP3-Neg) LUAD tissues (Figure 6H). These findings highlight that MCPIP3 and IGF2BP3 may mediate METAP2 expression in human lung cancer tissues in negative and positive manners, respectively. We also found that the expression of many metastasis-promoting genes was significantly associated with the expression of MCPIP3 and IGF2BP3 in LUAD tissues (Figure S6D). Based on these results, we proposed a model to describe the potential antagonistic effects of the RBPs MCPIP3 and IGF2BP3 in regulating lung cancer cell metastasis (Figure 6I). MCPIP3, which is expressed at a relatively low level in human lung cancer cells, destabilizes METAP2 mRNA by binding to the stem–loop structure in the 3′UTR via its RNase domain, thereby downregulating METAP2 gene expression and inhibiting lung cancer cell metastasis. Conversely, the high expression of IGF2BP3 in lung cancer cells stabilizes METAP2 mRNA by targeting the common stem–loop structure, thereby promoting METAP2 gene expression as well as lung cancer metastasis (Figure 6I). In summary, these findings strongly suggest that MCPIP3 and IGF2BP3 exert distinctly opposite effects on lung cancer metastasis progression by antagonistically regulating METAP2 gene expression. In the future, effectively inducing MCPIP3 while simultaneously inhibiting IGF2BP3 expression in lung cancer cells may represent a promising strategy for controlling lung cancer metastasis.

## 4. Discussion

To date, the expression patterns of RBPs in human lung cancer tissues remain unclear. This study revealed the expression of all the RBPs in LUAD and LUSC tissues. We found that the expression level of the RBP MCPIP3 was significantly downregulated in both LUAD and LUSC, whereas IGF2BP3 expression was significantly increased. Both RBPs were found to influence the metastasis progression of human lung cancer. In contrast, MCPIP3 significantly inhibited lung cancer cell metastasis, whereas IGF2BP3 promoted cancer cell metastasis. It should be pointed out that the limited detection of lung cancer cell lines might be one of the limitations of this study. However, A549 and NCI-H23 are typical lung adenocarcinoma cell lines and non-small-cell lung cancer cell lines, respectively, indicating that the two RBPs can play a role in different types of lung cancer. Both of these RBPs were able to regulate the expression of metastasis-promoting genes in human lung cancer cells. To the best of our knowledge, few studies have reported the antagonistic regulatory effects of these two RBPs on the expression of tumor metastasis-related genes, especially in human lung cancer cells. Therefore, our findings revealed a reversible regulatory mechanism mediated by RBPs in controlling the metastasis of human lung cancer cells. In the future, the impacts of these two RBPs on human lung cancer metastasis might be further validated through patient-derived models, such as PDX (patient-derived xenograft) experiments.

METAP2 is a well-known tumor metastasis promoter involved in regulating the metastasis progression of various human cancers and is considered a promising therapeutic target for cancers [35,36]. To date, few studies have reported the molecular mechanisms regulating METAP2 gene expression, especially in human lung cancer cells. In this study, we discovered that RBP MCPIP3 downregulates METAP2 expression by degrading its mRNA through its RNase domain. Our results revealed the post-transcriptional regulation mechanism of METAP2 gene expression in human lung cancer cells and further elucidated the downstream targets of the MCPIP protein family. We also revealed that MCPIP3 preferentially binds to metastasis-promoting mRNAs rather than metastasis-suppressing mRNAs. This preference may result from the long-term evolution of cancer cells; however, further investigations are required to clarify its precise underlying mechanism.

The RNA secondary stem–loop structure located in the 3′UTR is believed to be an important *cis*-acting element that can be recognized and bound by RBPs to control transcript stability [37,38]. It is therefore not surprising that MCPIP3 binds to the stem–loop structure in the 3′UTR of METAP2 mRNA, as previous studies have shown that other family members of MCPIPs also recognize stem–loop RNA [39]. Interestingly, this study further revealed that IGF2BP3, another RBP, was also capable of binding to the same structural RNA element but instead stabilized METAP2 mRNA and promoted its expression in human lung cancer cells. It has been reported that IGF2BP3 stabilizes mRNA [40] and promotes lung cancer metastasis [34], which is consistent with our findings. Previous data from our laboratory and other groups have shown that IGF2BP1, a homolog of IGF2BP3, can bind to structural RNA [41,42], further supporting our findings. In this study, our findings revealed a novel regulatory mechanism, in which IGF2BP3 and MCPIP3 antagonistically regulate the stability and expression of the metastasis-promoting METAP2 gene transcript by targeting a common stem–loop structure in human lung cancer cells. These results provide new insights into the understanding of the regulatory mechanisms controlling METAP2 gene expression in cancer cells.

Our findings suggest an adaptive regulatory behavior of lung cancer cells. Specifically, we found that MCPIP3 can inhibit lung cancer cell metastasis but that its expression is suppressed. However, the expression of IGF2BP3 is increased and promotes metastasis. One consequence of this is that lung cancer cells can increase the expression of the metastasis-promoting gene METAP2, ultimately facilitating lung cancer cell metastasis. These findings lead us to speculate that RBPs in human lung cancer cells may form a “yin” and “yang” regulatory mechanism, which antagonistically or synergistically regulates the expression of tumor-related genes in cancer cells. Further investigation is still necessary to elucidate the molecular mechanisms through which RBPs regulate cancer progression and the potential upstream regulators or signaling pathways that may maintain the homeostatic balance between MCPIP3 and IGF2BP3 during lung cancer progression.

## 5. Conclusions

In conclusion, the results of this study reveal that the RBPs MCPIP3 and IGF2BP3 antagonistically regulate the mRNA stability and expression of the metastasis-promoting gene METAP2 by binding to a common stem–loop structure, leading to reversible regulation of human lung cancer metastasis. These results provide new insights into the regulatory mechanisms of human lung cancer metastasis and identify potential molecular targets for the future treatment of human lung cancer metastasis.

## Data Availability

All data supporting the findings of this study are included in the article and/or the Supplementary Materials. The RNA sequencing data from this study are deposited in the Sequence Read Archive (SRA) database under projects PRJNA1119197 and PRJNA1119237. The MS data are available via ProteomeXchange with the IDs: PXD052919 and PXD052920.

## Supplementary Materials

The following supporting information can be downloaded at: https://www.mdpi.com/article/10.3390/cancers18152384/s1, Figure S1: MCPIP3 expression is reduced in human lung cancer tissues; Figure S2: MCPIP3 regulates the expression of metastasis-related genes in human lung cancer cells; Figure S3: MCPIP3 selectively downregulated the expression of the metastasis-promoting gene METAP2 by destabilizing its mRNA via the RNase domain; Figure S4: MCPIP2 depletion increased proangiogenic mRNAs stability and promoted tumor angiogenesis; Figure S5: IGF2BP3 interacts with the common stem–loop structure to stabilize METAP2 mRNA and promote lung cancer metastasis; Figure S6: IGF2BP3 and MCPIP3 potentially antagonize metastasis-promoting gene expression and lung cancer cell metastasis; Table S1: PCR primers and RNA-EMSA probe sequences; Table S2: PCRarray analysis of tumor metastasis-related genes regulated by MCPIP3; Table S3: RNA-seq analysis of genes regulated by MCPIP3 or IGF2BP3 in human lung cancer cells; Table S4: Identifying proteins that interact with the METAP2 RNA stem–loop structure in A549 cells using mass spectrometry; File S1: Supplementary Materials and methods; File S2: Original images.
