# Supplementary material for: RNA-Binding Proteins MCPIP3 and IGF2BP3 Antagonize Lung Cancer Metastasis by Reversibly Regulating METAP2 Expression via mRNA Stability
Source: Cancers (Basel). 2026 Jul 24;18(15):2384. doi: 10.3390/cancers18152384 (PMC13465609; doi:10.3390/cancers18152384)
Supplement: Supplementary file 1 [file cancers-18-02384-s001.zip › cancers-4393166-File S1.pdf]

File S1: Supplementary materials and methods

Bioinformatic analysis of RBPs expression in human LUAD and LUSC datasets

Human LUAD and LUSC datasets were downloaded from the TCGA (https:// tcga-data.nci.nih.gov/tcga/). The gene expression matrix was selected only from the primary tumors and their adjacent health tissues. The known 1756 RNA-binding proteins genes were analyzed. The limma package of R studio (version 4.0.0) was used for differential analysis of target genes (cutoff: |log2FC|>1 & FDR<0.05).

Antibodies and reagents

Polyclonal rabbit anti-MCPIP3 (PA5-20928) was from Invitrogen (Grand Island, USA), anti-METAP2 (17040-1-AP), anti-IGF2BP3 (14642-1-AP) was from Proteintech Co. (Wuhan, China), and anti-GFP (A-11122) was from ThermoFisher (Waltham, MA, USA). Isotype normal rabbit IgG (ab37415) was from Abcam (Cambridge, UK). Mouse GAPDH (A19056) and β-actin (AC004) antibodies were from ABclonal Company (Beijing, China). G418 (G8168), puromycin (P8833), Actinomycin D (ActD, A1410), and 5, 6-Dichlorobenzimidazole 1-β-D-ribofuranoside (DRB, D1916) were from Sigma-Aldrich (St. Louis, USA). Protein A/G PLUS-Agarose beads were from Sant Cruz Biotechnology (San Diego, CA, USA). GFP-coated beads (gta-20) were from ChromoTek (Munich, Germany). Dynabeads M-280 streptavidin (11205D) were from Invitrogen.

Gene Ontology analysis

The commonly upregulated and downregulated mRNAs by MCPIP3 or IGF2BP3 in human lung cancer cells were classified by the Venn diagram online tool. GO analyses of differentially expressed genes were performed using DAVID (http://david.abcc.nifcrf.gov/).

RNA immunoprecipitation (RIP)

A549 cells expressing MCPIP3-GFP or empty vector were lysed and the whole cell lysates precleared with IgG, and then incubated with GFP-coated beads (GFP-trap beads) for 4 hours at 4°C. The RNA-protein complexes were pulled down by GFP-trap beads. The precipitated mRNAs were extracted from the beads and reverse transcribed to cDNA for RT-PCR detection.

RNA immunoprecipitation-chromatin immunoprecipitation (RIP-ChIP)

Cultured A549/MCPIP3-GFP cells were cross-linked for 10 min by formaldehyde (1% v/v). Glycine was added to stop the crosslinking (125 mM). Cells were washed with cold PBS and resuspended in 500 μL of polysome lysis buffer, and then placed on ice for 5 min. Cell lysates were collected by centrifugation and re-suspended in 500 μL of polysome lysis buffer. Then, the lysates were sonicated and pre-cleared with isotype IgG to remove non-specific background. Pre-cleared lysates were used for IP with GFP-coated beads or IgG-coated beads at 4°C for 4 h. After pull-down, 100 μL supernatants were taken out for Input. The precipitated complexes were washed at least five times with ice-cold NT2 buffer. RNA was isolated with Trizol, and re-suspended in 50 μL of RNase-free water, followed by DNase I treatment and further detection.

Luciferase reporter assays

Luciferase assay was performed as described previously. The full-length or segment of 3'UTR of the indicated genes were amplified by PCR and cloned into the pGL3 luciferase reporter. HEK293 cells were then transfected with MCPIP3, IGF2BP3, truncations, and GFP-control constructs, respectively. After transfection for 36 hours, the luciferase activity was measured using a Dual-Luciferase Reporter Assay System (Promega, USA). All transfections were conducted in triplicate and repeated at least three times.

shRNA lentivirus

Lentiviral shRNAs targeting human *MCPIP3* (TRCN0000237943; TRCN0000237946) and *METAP2* mRNA (TRCN0000286742) were purchased from Sigma. A scramble control shRNA was used as a control. Lentiviral particles were packaged by co-transfecting shRNA-pLKO.1, pCMV-dR8.2, and pMD2.G constructs in HEK293T cells. After two rounds infection, the target cells were selected with puromycin (1.0μg/mL) for two weeks. IGF2BP3/GFP-overexpressing lentivirus, MCPIP3/GFP-overexpressing lentivirus were packaged by GeneChem Company (Shanghai).

Tumor tissue samples

A total of ten human LUAD tissue samples and their matched surrounding ‘healthy’ tissues were obtained between April 2024 and November 2025. Among these samples, five samples were assessed as MCPIP3 high expression (MCPIP3-positive) and five samples showed MCPIP3 low expression (MCPIP3-negative) according to IHC scoring. Six samples were

evaluated as IGF2BP3 high expression (IGF2BP3-positive) and four samples showed lower IGF2BP3 expression (IGF2BP3-negative). All subjects gave their informed consent for inclusion before they participated in this study. The study was conducted in accordance with the Declaration of Helsinki, and the experimental protocols were approved by the Medical Ethics Committee of the Institute of Microcirculation, CAMS & PUMC.

Immunohistochemistry (IHC) and hematoxylin & eosin (H&E) staining

Xenograft tumor tissues or human lung cancer tissues and adjacent non-cancerous tissues were subjected to IHC to detect the expression of interested proteins with indicated antibodies. Whole lung of nude mice was first immersed in 10% formaldehyde solution for two weeks and then treated with H&E for staining. Leica Imaging System was used to visualize the staining sections.

Sequence alignments and stem–loop structure prediction

The 3'UTR sequences of *METAP2*, *HRAS*, *PNN*, and *CTBP1*, were extracted for different species from the National Center for Biotechnology Information (NCBI) database: *METAP2* 3'UTR: human (*Homo sapiens*; accession number NM_006838.4), chimpanzee (*Pan troglodytes*; NM_001280056.1), mouse (*Mus musculus*; NM_019648.3), rat (*Rattus*; NM_022539.2); *HRAS* 3'UTR: human (*Homo sapiens*; NM_001130442.3), chimpanzee (*Pan troglodytes*; XM_054661834.1), mouse (*Mus musculus*; NM_001412481.1), rat (*Rattus*; NM_001098241.1); *PNN* 3'UTR: human (*Homo sapiens*; NM_002687.4), chimpanzee (*Pan troglodytes*; XM_001147812.7), mouse (*Mus musculus*; NM_008891.2), rat (*Rattus*; XM_032907790.1); *CTBP1* 3'UTR: human (*Homo sapiens*; NM_001377192.1), chimpanzee (*Pan troglodytes*; XM_054683688.2), mouse (*Mus musculus*; NM_001198859.1), rat (*Rattus*; NM_019201.3). Conserved stem–loop sequences analysis was performed using DNAMAN software and predicted through RNAfold web server (http://rna.tbi.univie.ac.at/).
