# Supplementary material for: RNA-Binding Proteins MCPIP3 and IGF2BP3 Antagonize Lung Cancer Metastasis by Reversibly Regulating METAP2 Expression via mRNA Stability
Source: Cancers (Basel). 2026 Jul 24;18(15):2384. doi: 10.3390/cancers18152384 (PMC13465609; doi:10.3390/cancers18152384)

Figure 1E

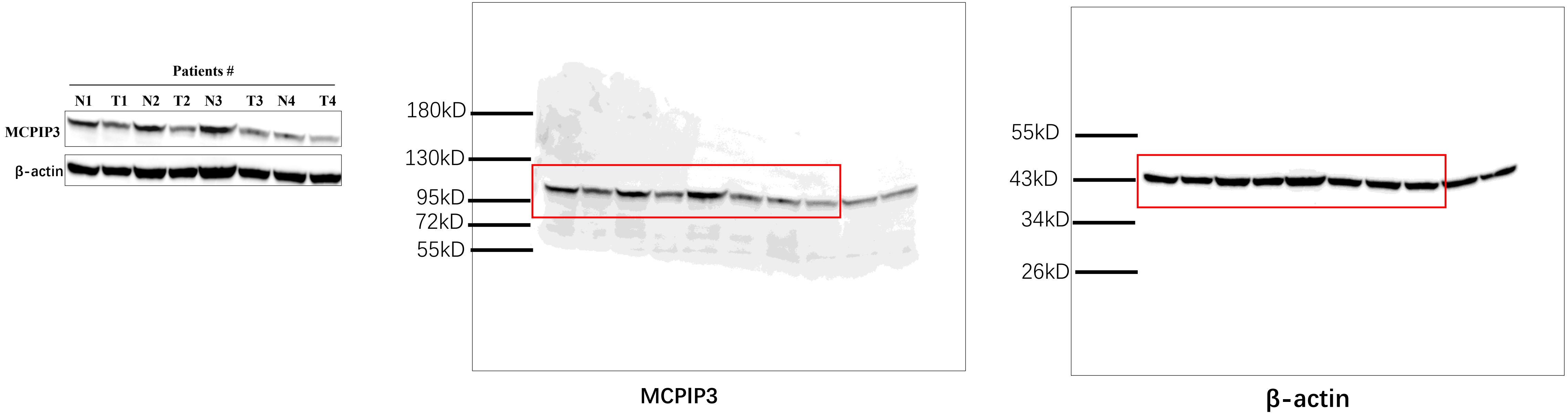

Figure 1F

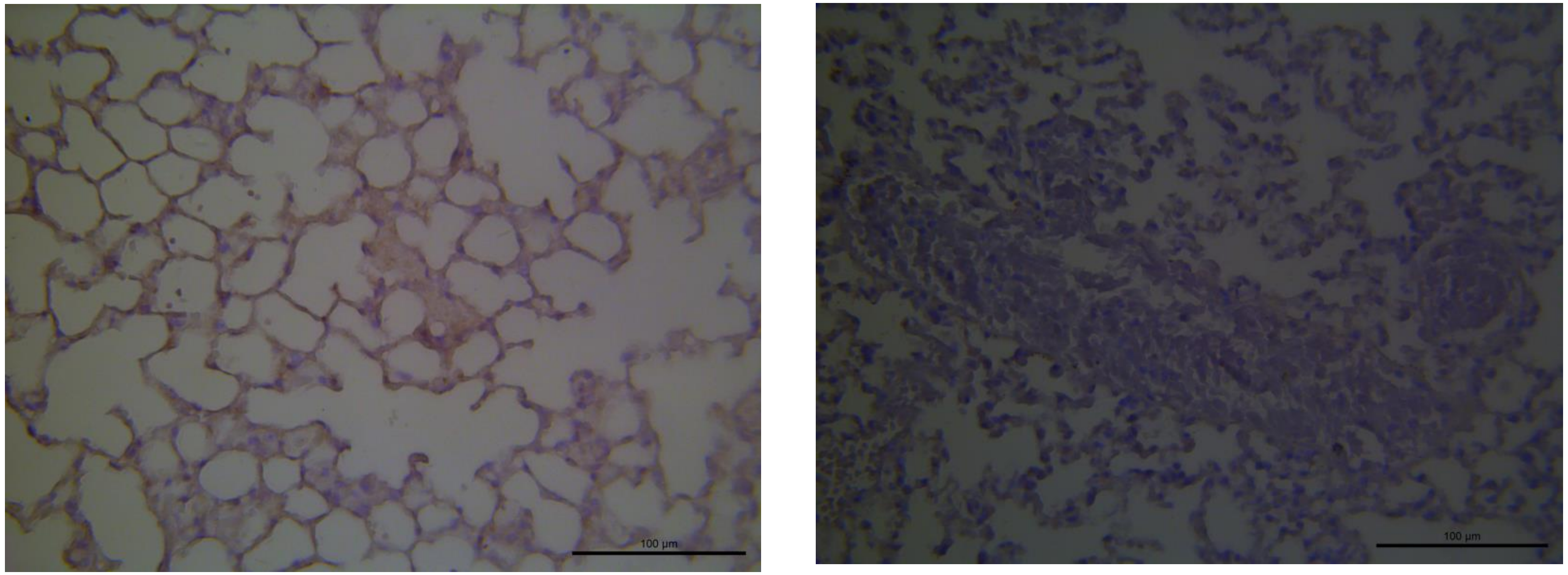

Figure. 2A

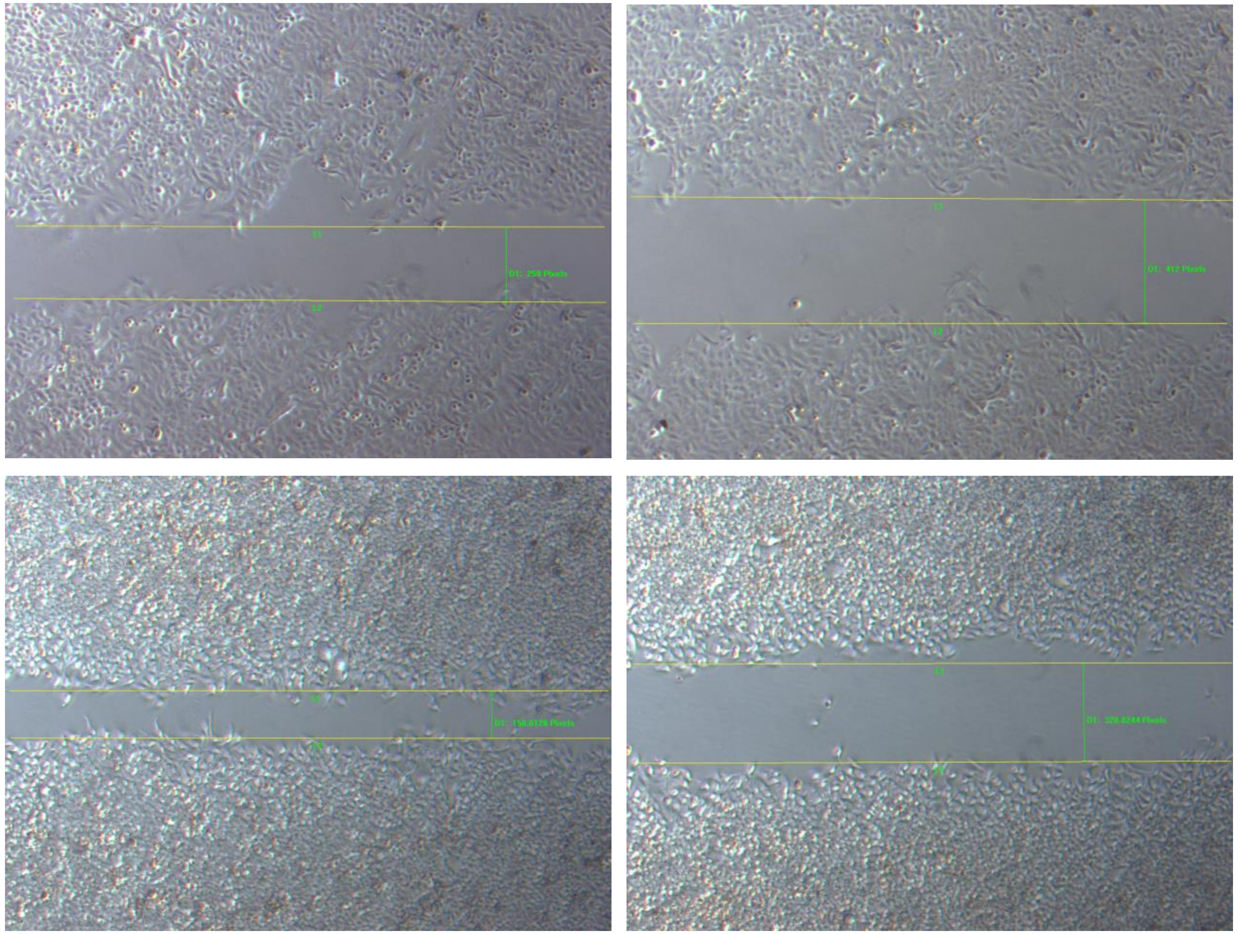

Figure. 2E

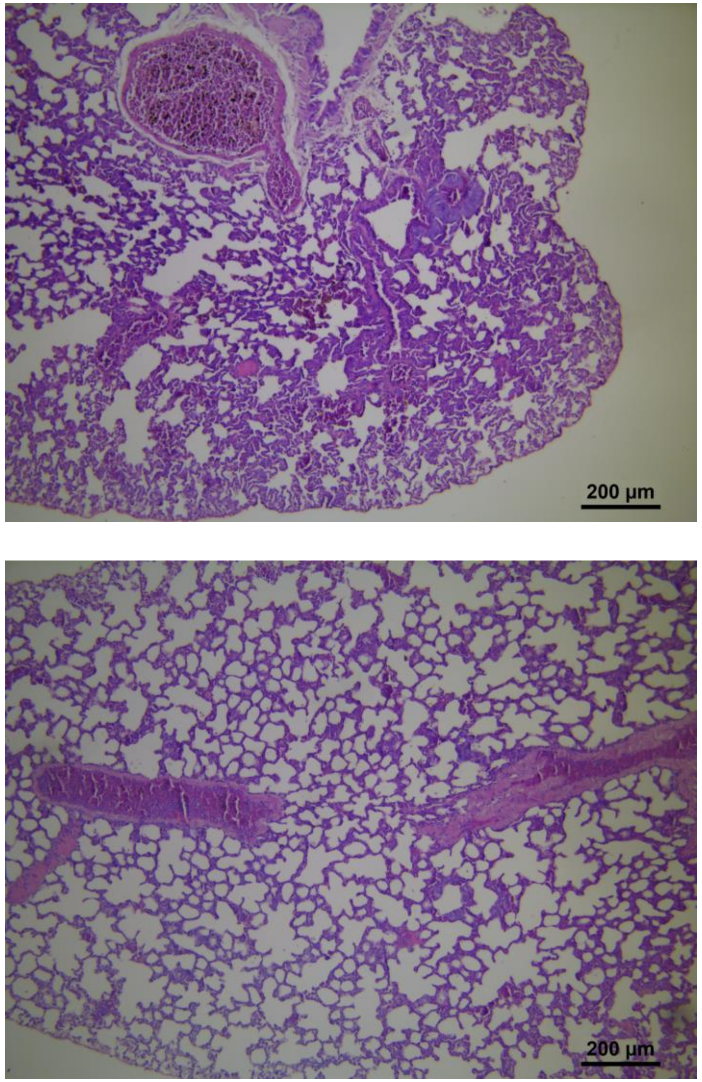

Figure. 3A

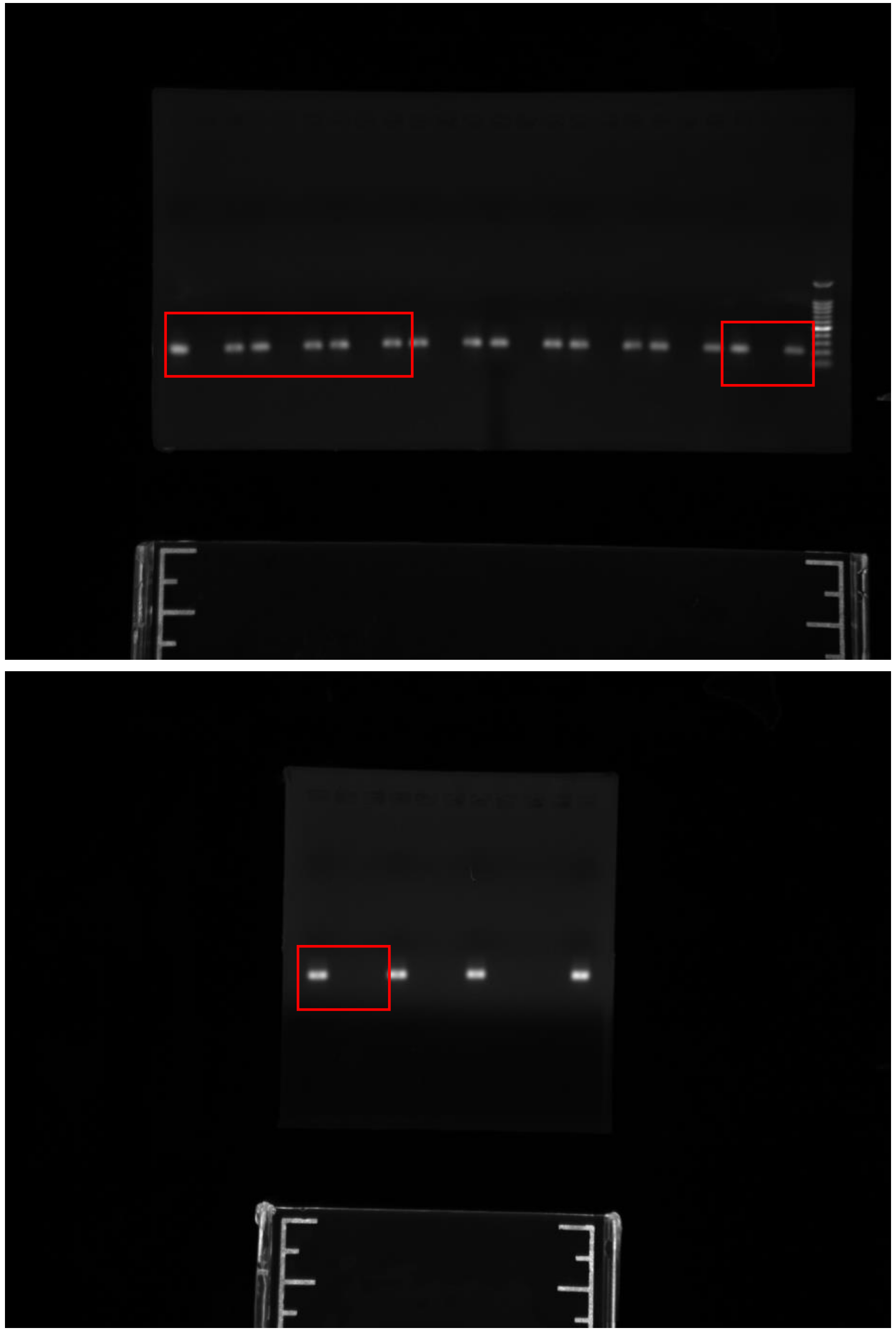

Figure. 3E

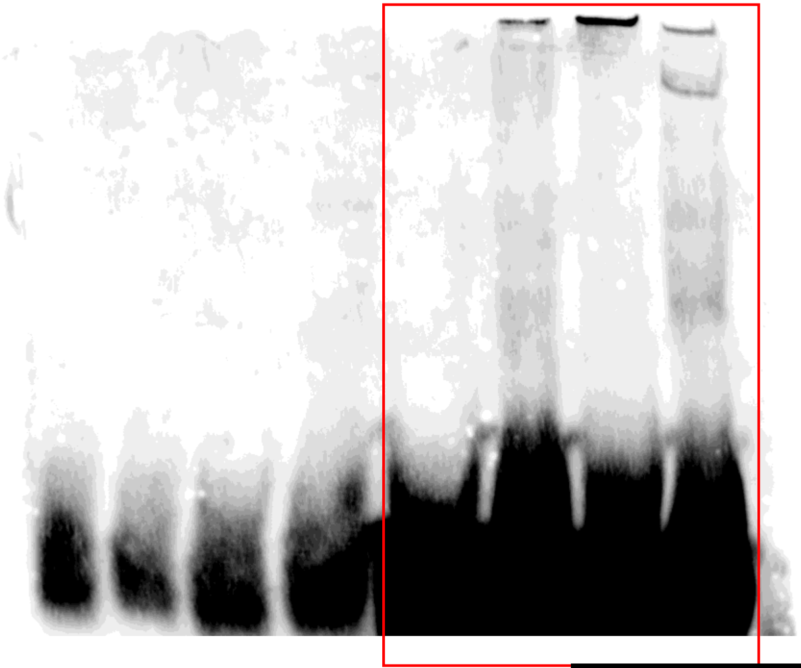

Figure. 3F

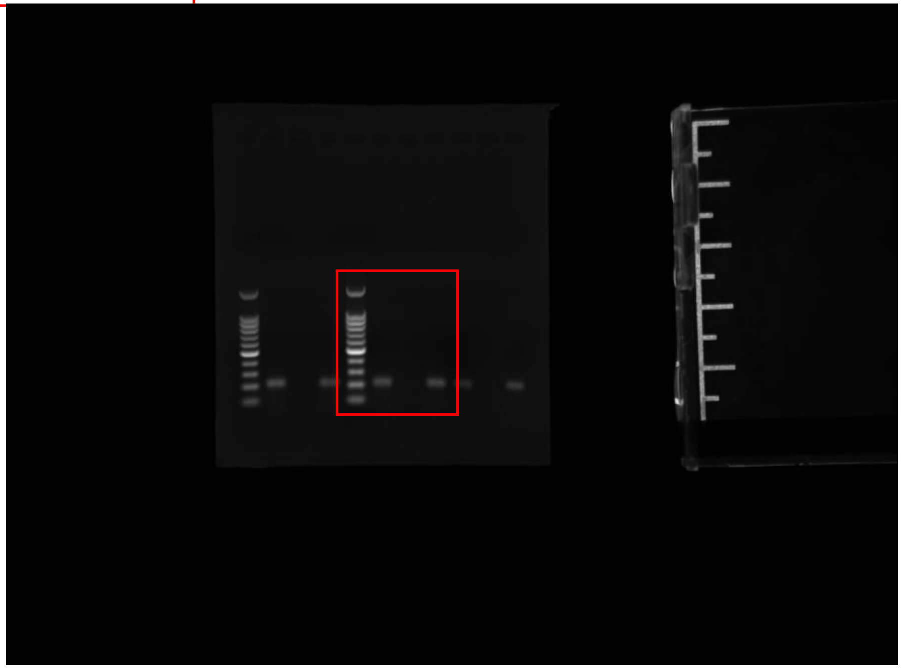

Figure. 3H

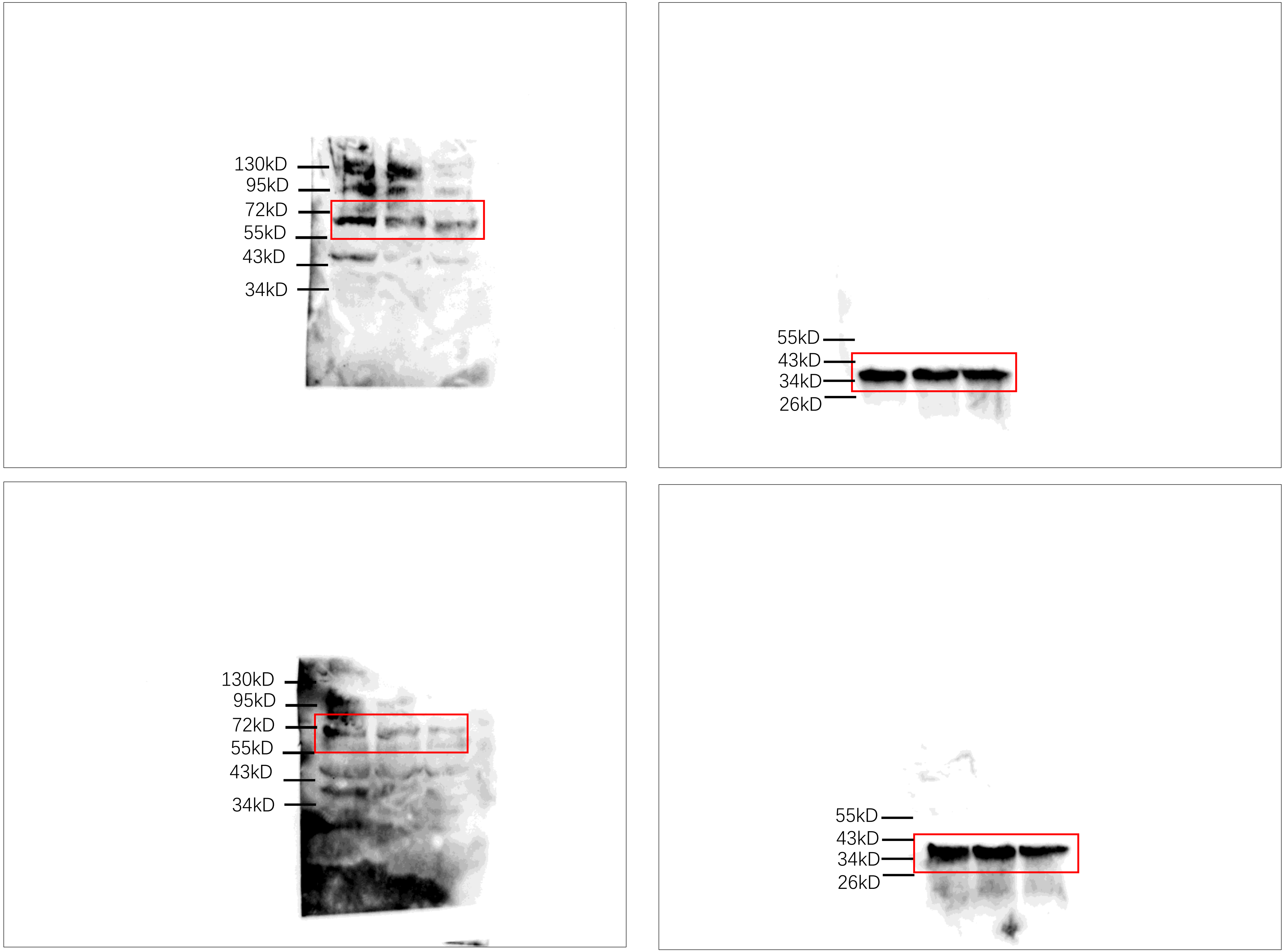
130kD
95kD
72kD
55kD
43kD
34kD
55kD
43kD
34kD
26kD
130kD
95kD
72kD
55kD
43kD
34kD
55kD
43kD
34kD
26kD

Figure. 4A

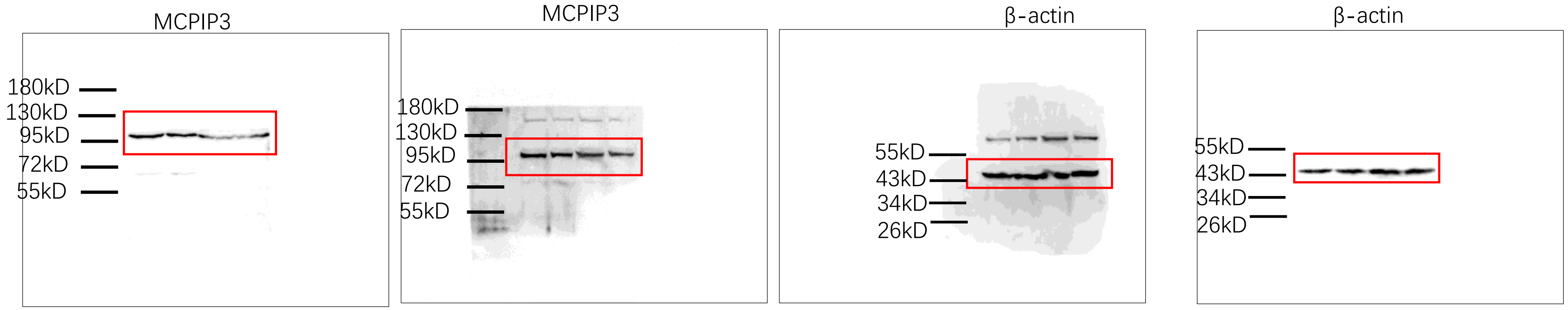

Figure. 4B

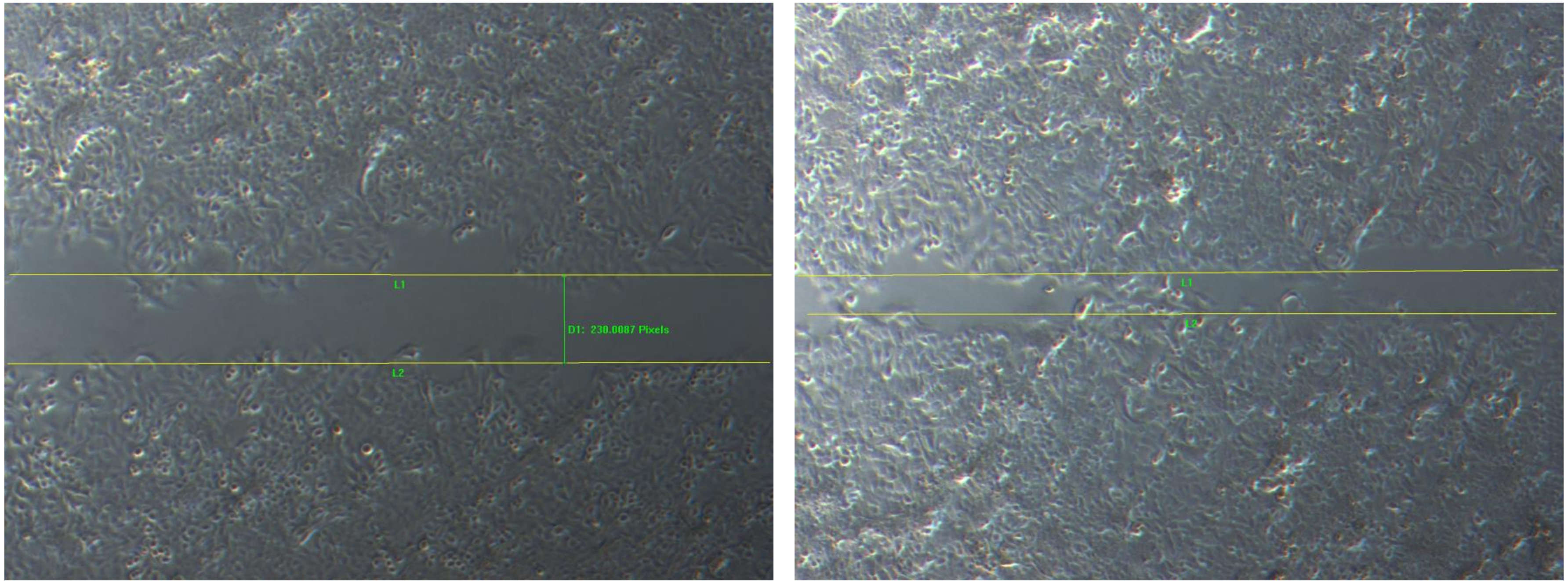

Figure. 4B

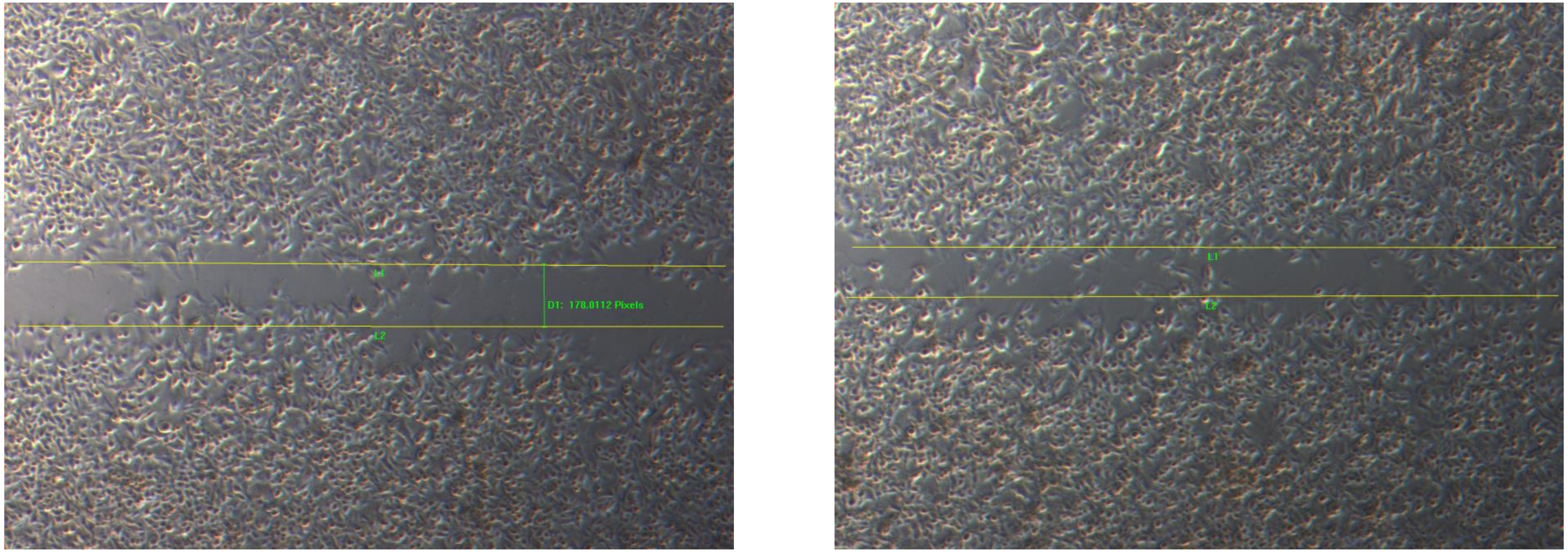

Figure. 4I

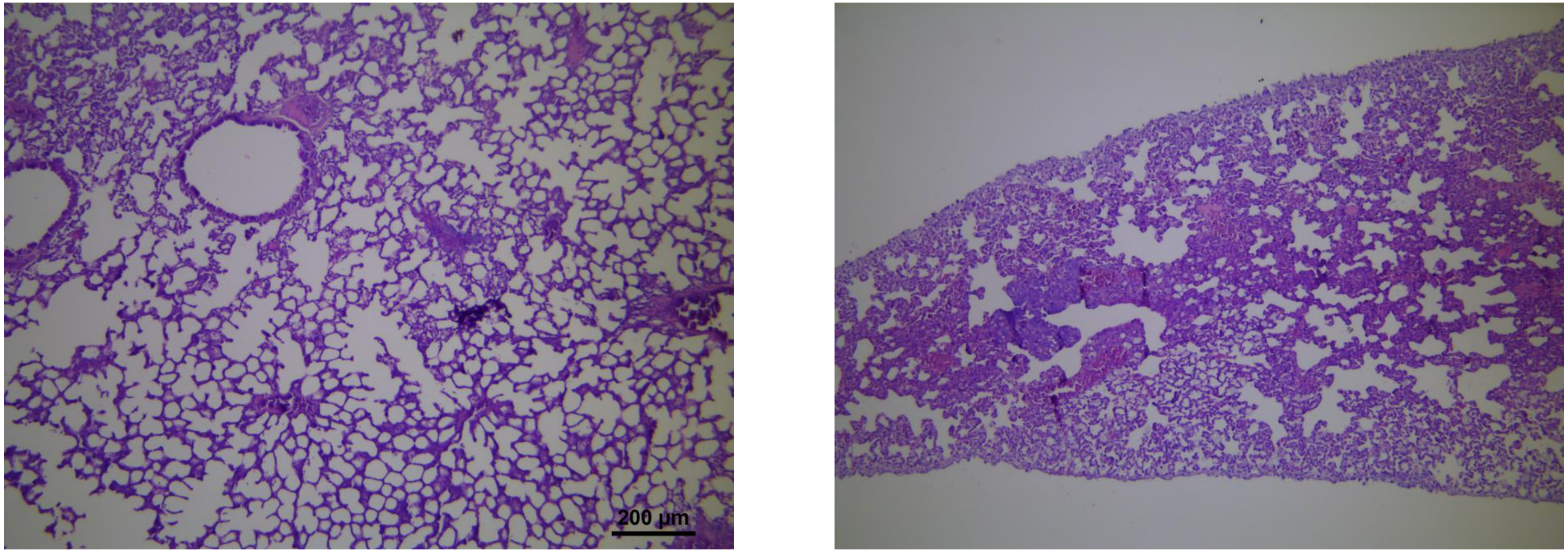

Figure. 5D

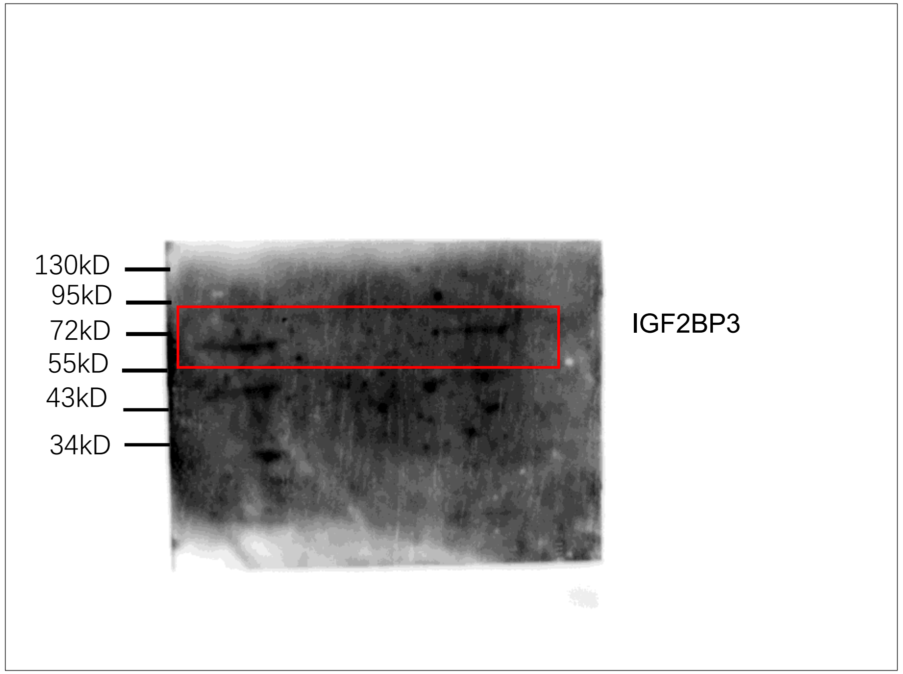

130kD
95kD
72kD
55kD
43kD
34kD
IGF2BP3

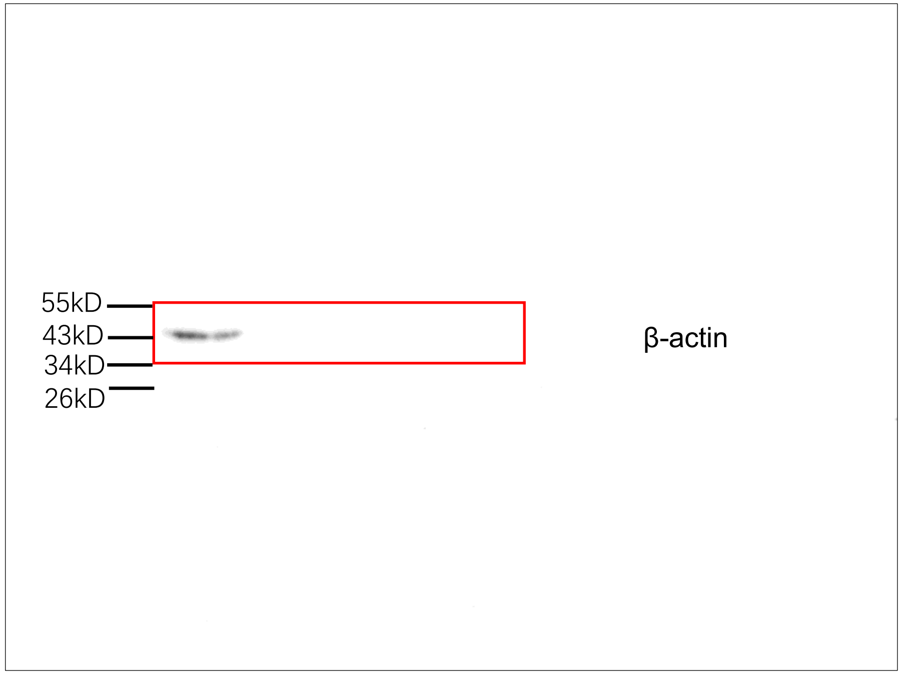

55kD
43kD
34kD
26kD
β-actin

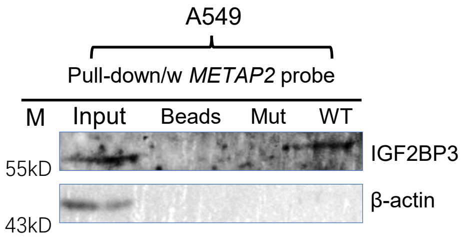

A549
Pull-down/w *METAP2* probe
M
Input
Beads
Mut
WT
55kD
43kD
IGF2BP3
β-actin

Figure. 5E

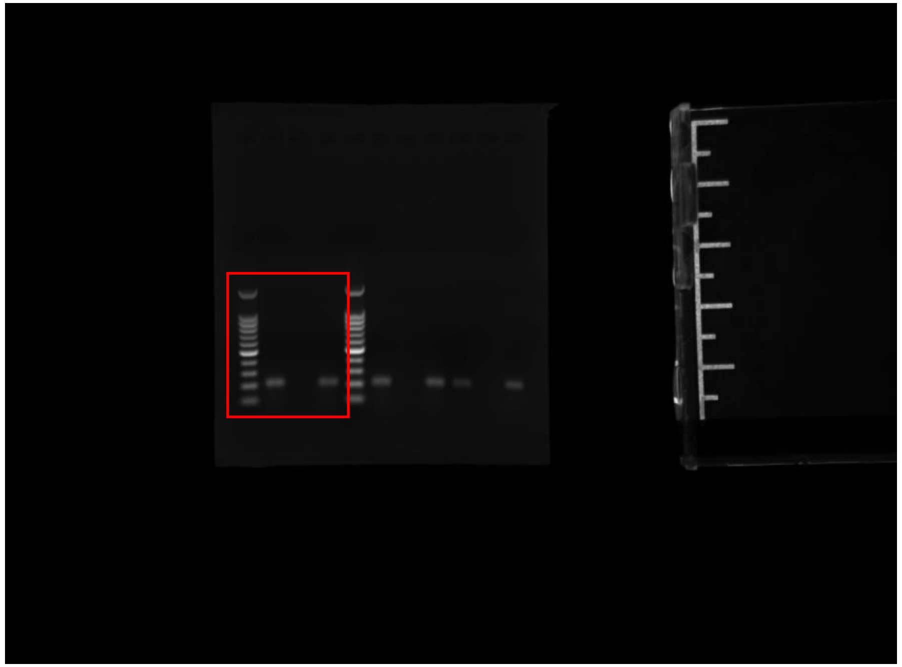

Figure. 5I

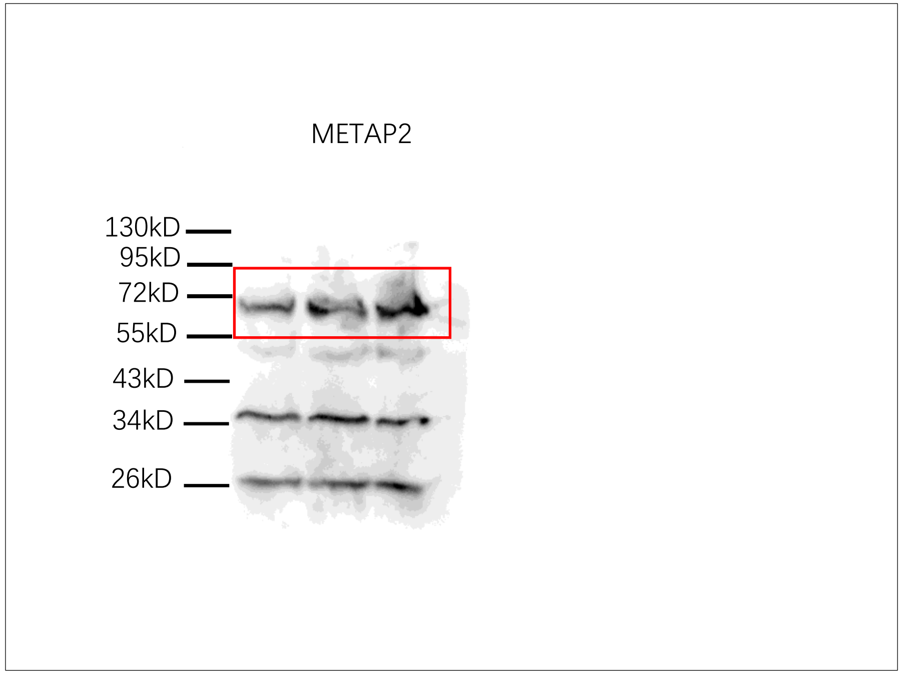
METAP2
130kD
95kD
72kD
55kD
43kD
34kD
26kD

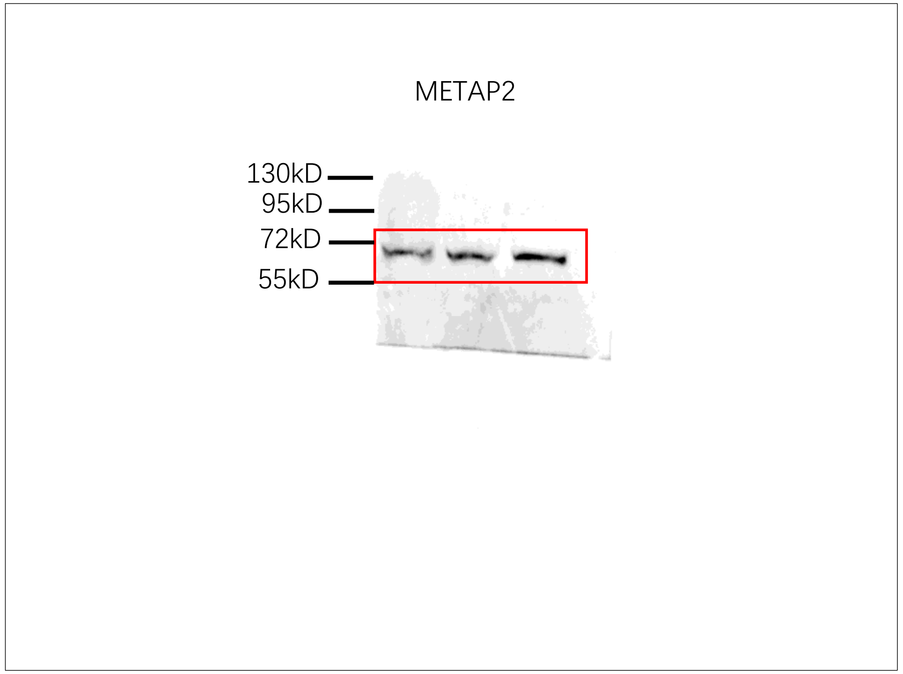
METAP2
130kD
95kD
72kD
55kD

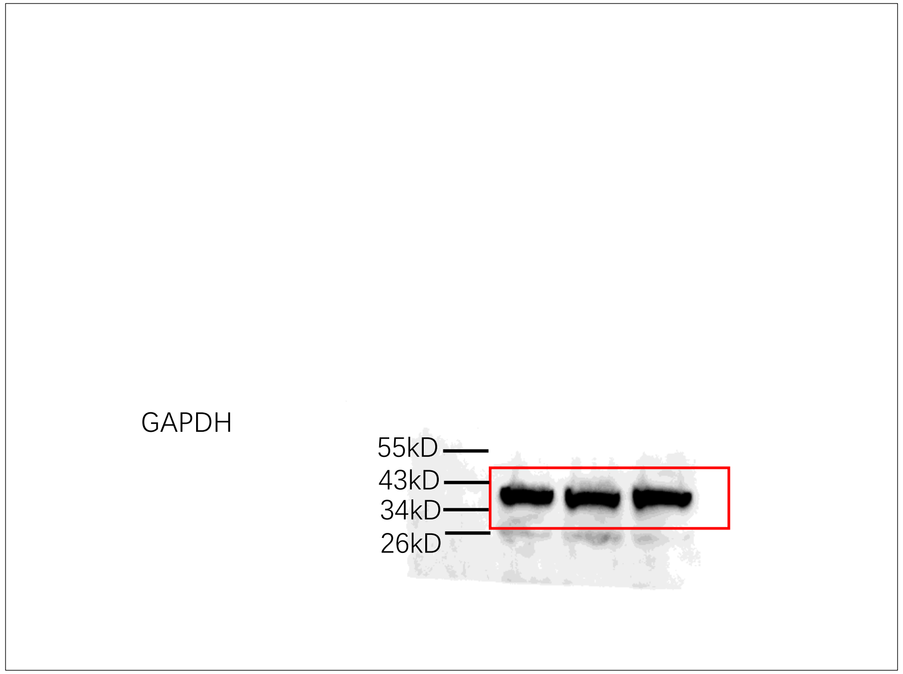
GAPDH
55kD
43kD
34kD
26kD

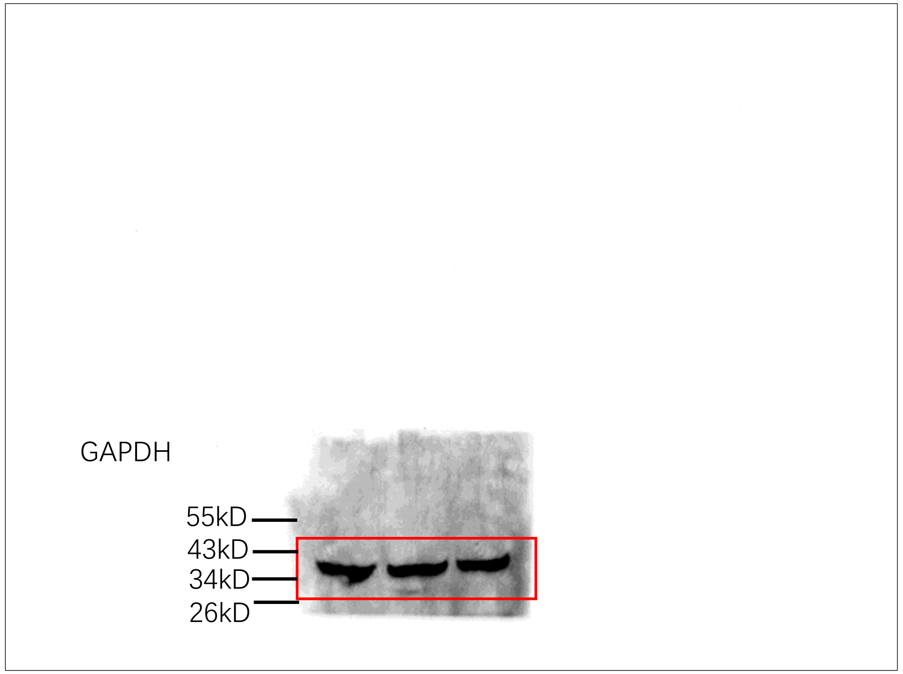
GAPDH
55kD
43kD
34kD
26kD

Figure. 5K

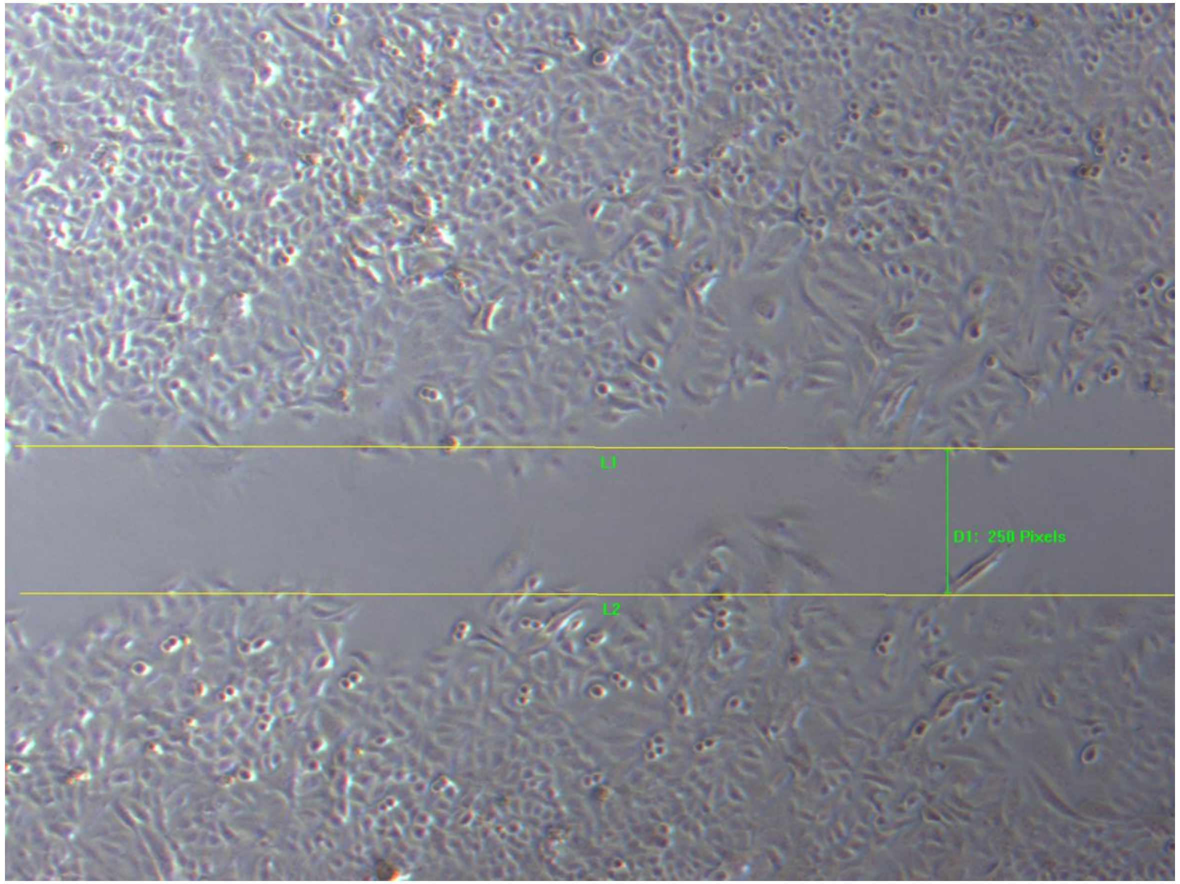

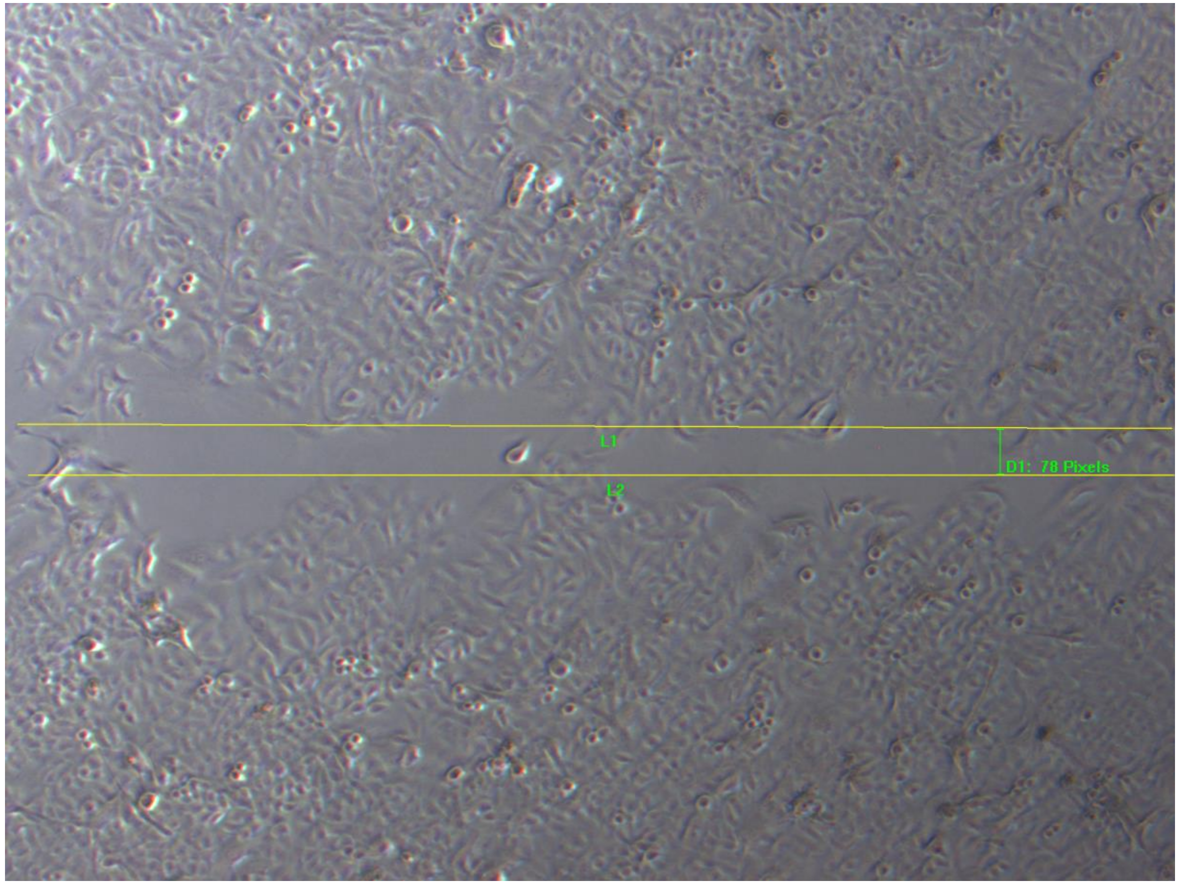

Figure. 6E

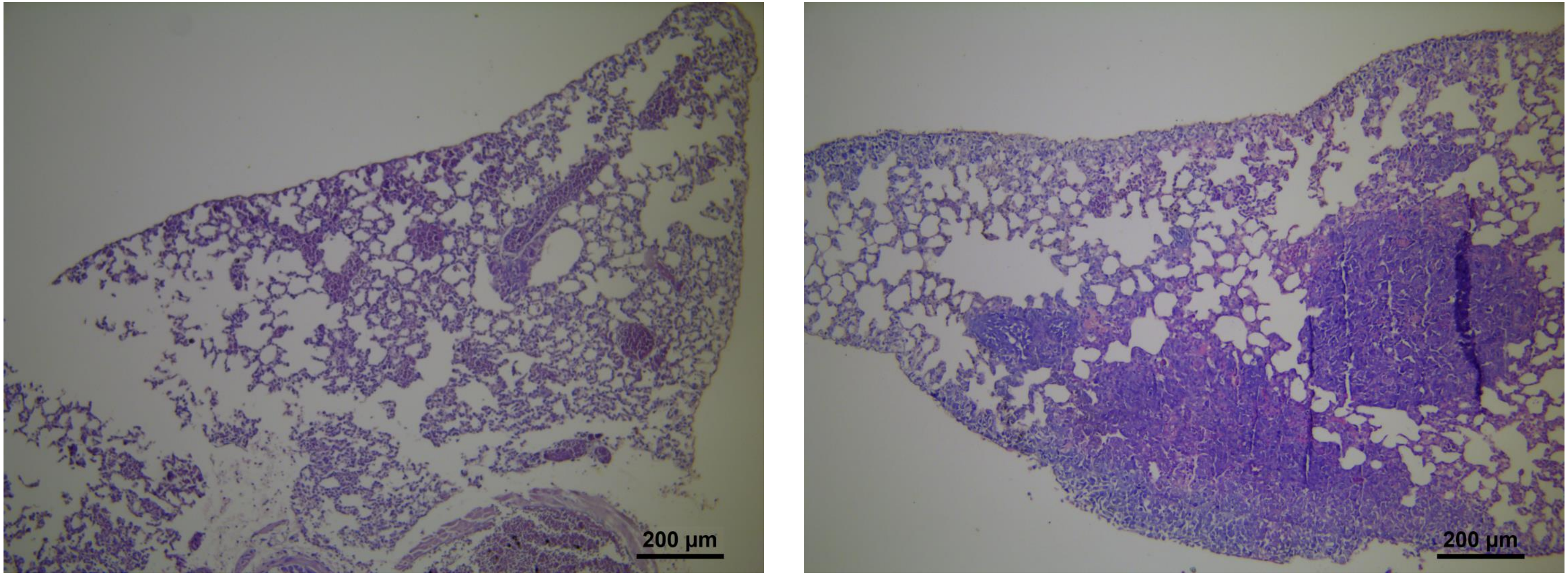

Figure. 6H

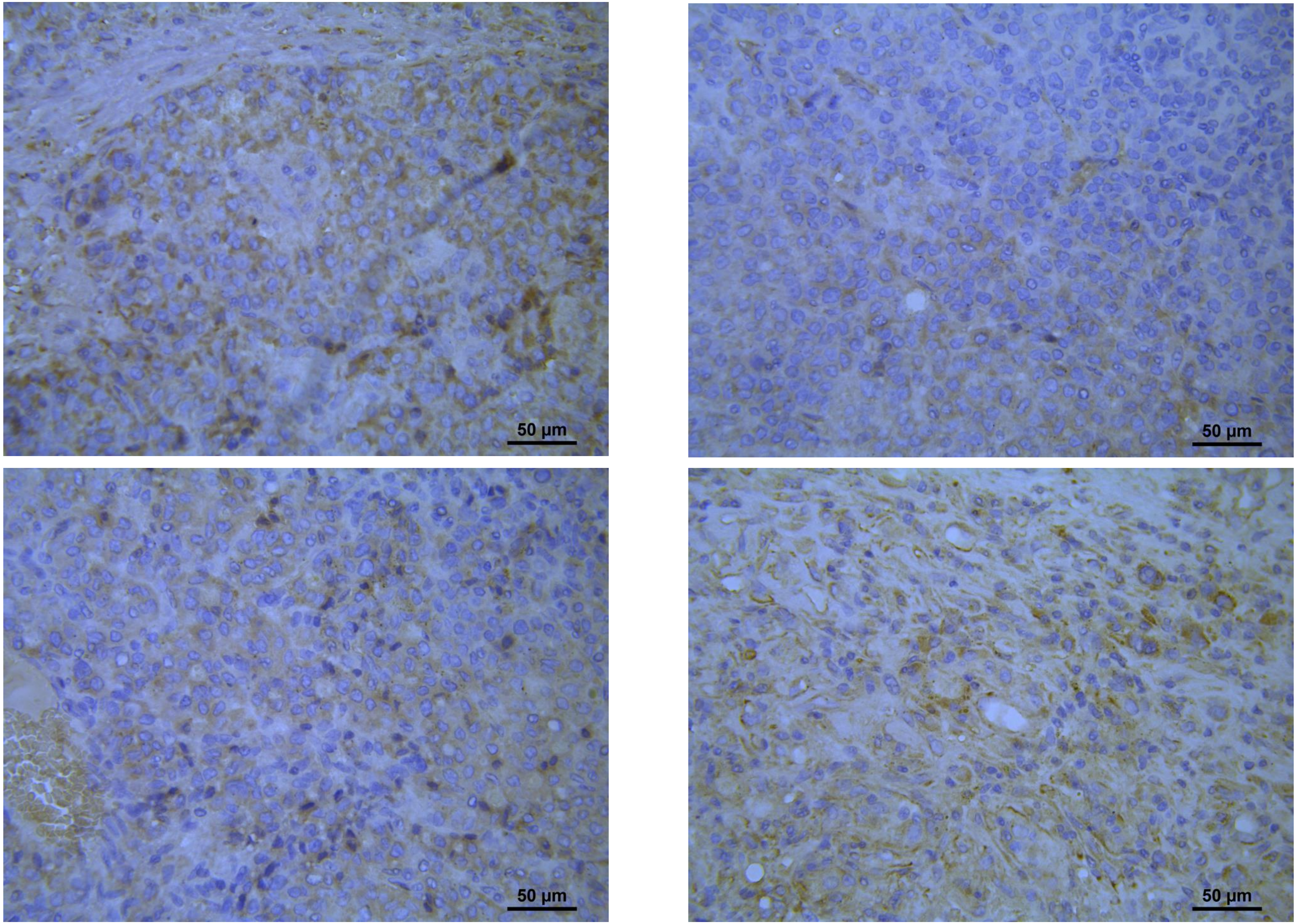

Figure. 6H

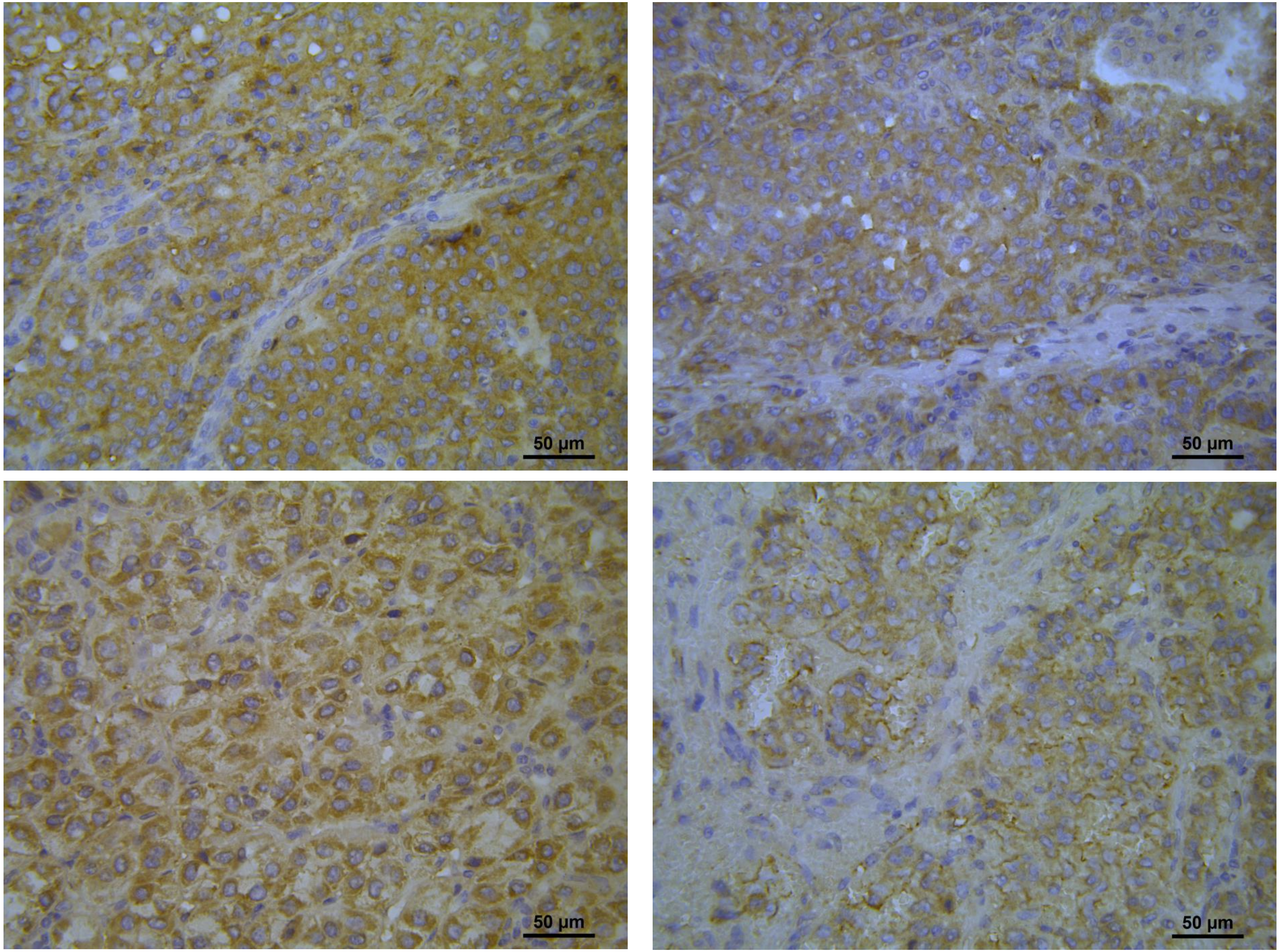

Figure.S2A

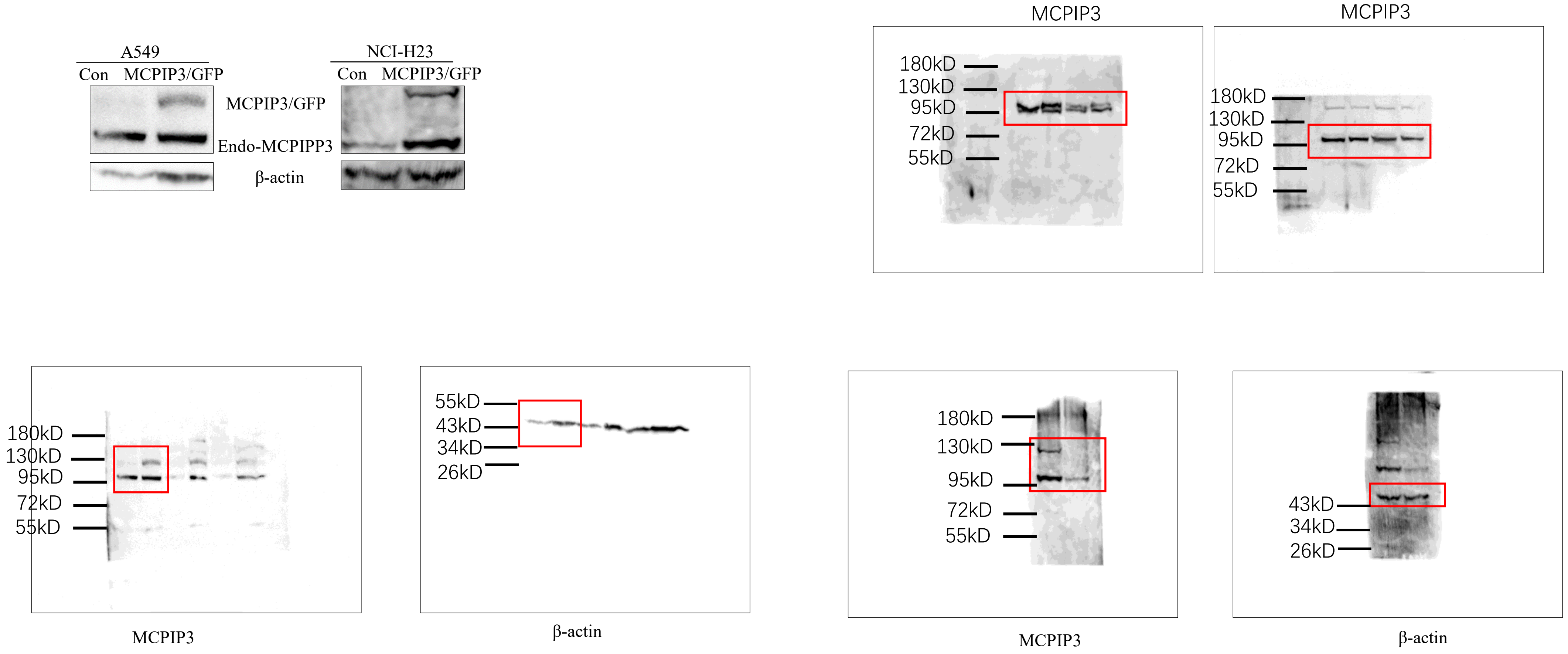
A549
Con MCPIP3/GFP
MCPIP3/GFP
Endo-MCPIPP3
β-actin
NCI-H23
Con MCPIP3/GFP
MCPIP3
180kD
130kD
95kD
72kD
55kD
MCPIP3
180kD
130kD
95kD
72kD
55kD
180kD
130kD
95kD
72kD
55kD
MCPIP3
55kD
43kD
34kD
26kD
β-actin
180kD
130kD
95kD
72kD
55kD
MCPIP3
43kD
34kD
26kD
β-actin

Figure.S3A

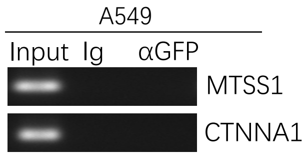

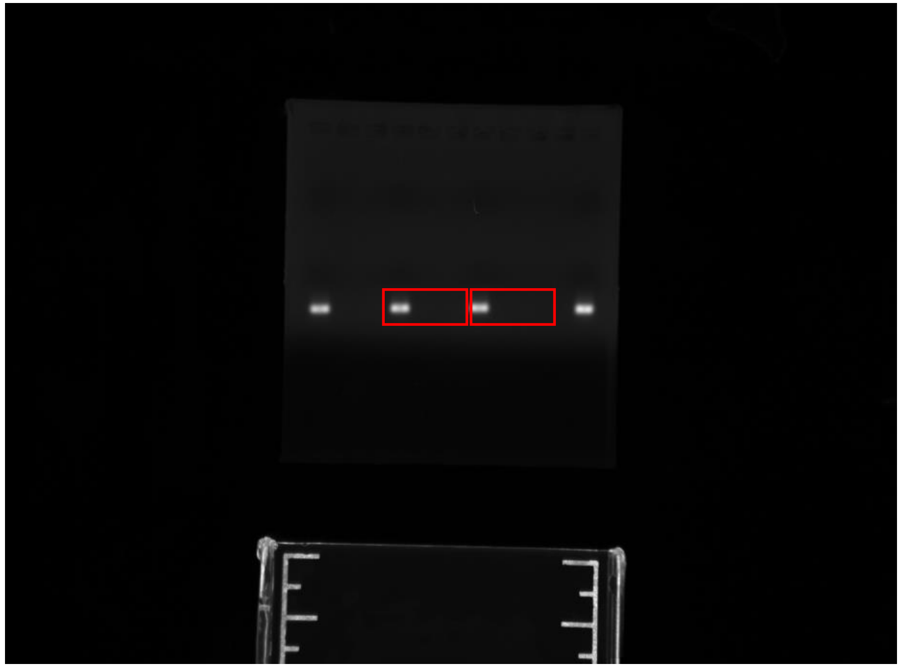

Figure.S4B

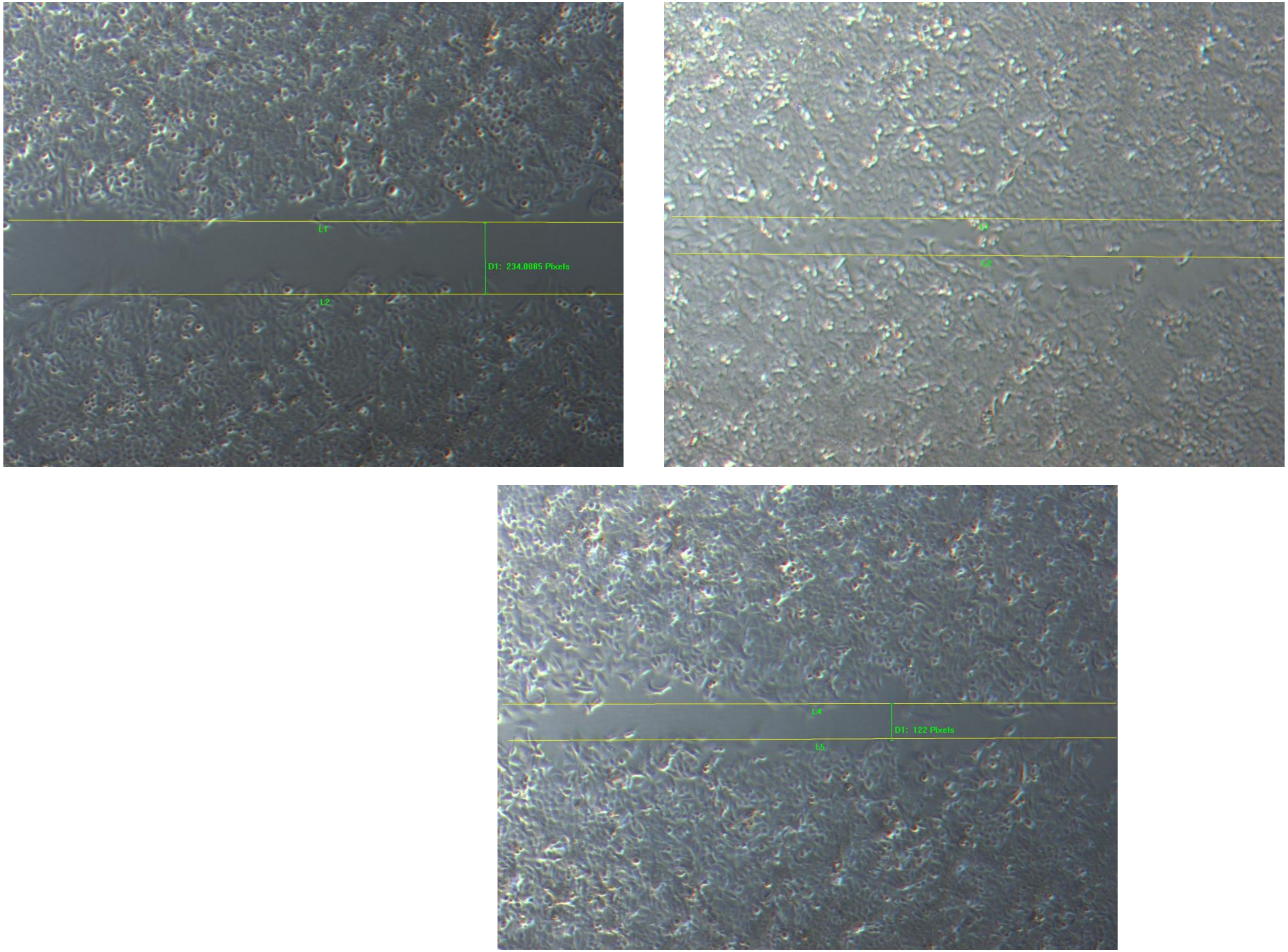

Supplement: Supplementary file 1 [file cancers-18-02384-s001.zip › cancers-4393166-file S2.pdf]
