# Supplementary material for: RNA-Binding Proteins MCPIP3 and IGF2BP3 Antagonize Lung Cancer Metastasis by Reversibly Regulating METAP2 Expression via mRNA Stability
Source: Cancers (Basel). 2026 Jul 24;18(15):2384. doi: 10.3390/cancers18152384 (PMC13465609; doi:10.3390/cancers18152384)

Figure S1. MCPIP3 expression is reduced in human lung cancer tissues. A The expression of 1,756 RNA-binding protein genes in human lung squamous cell carcinoma (LUSC) tissues was profiled according to the TCGA database. **B** The mRNA expression of MCPIP3 in human LUSC was analyzed by UALCAN online tool. **C** Expression of *MCPIP3* in lung cancer and other types of human cancers was analyzed by TIMER3 (https://compbio.cn/timer3/). **D** *MCPIP3* mRNA expression was reduced with the nodal metastasis status in LUSC patients (http://ualcan.path.uab.edu/analysis-prot.html). **E** *MCPIP3* mRNA expression was analyzed by the main pathological stages of LUSC.

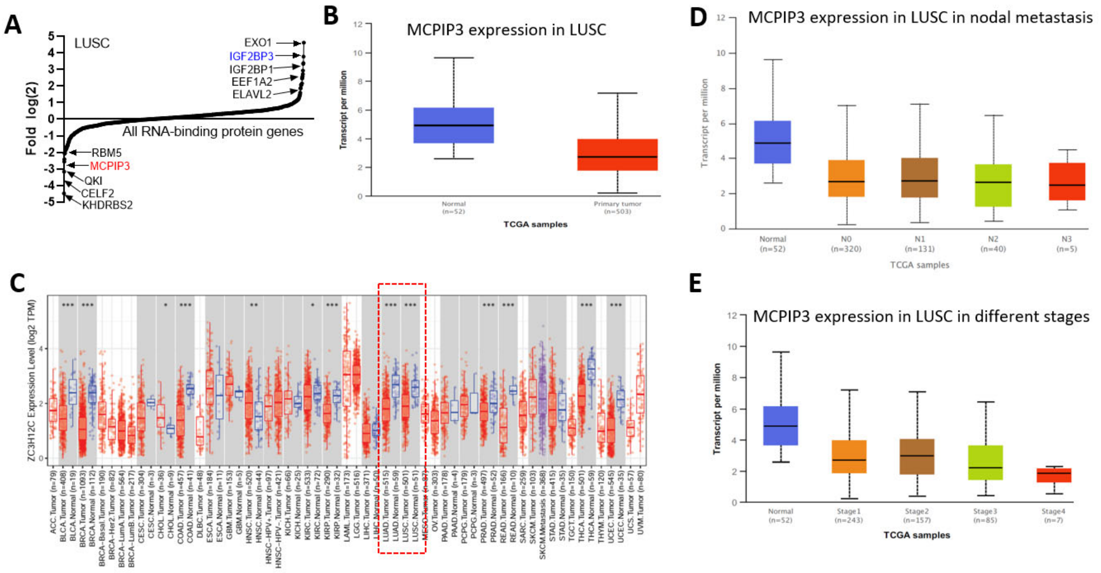

Figure S2. MCPIP3 regulates the expression of metastasis-related genes in human lung cancer cells. A Western blotting analysis of MCPIP3-GFP fusion protein expression in A549 and NCI-H23 cells with specific MCPIP3 antibody. **B** Venn diagrams showing the commonly up-regulated genes and down-regulated genes by MCPIP3 in human lung cancer cells. **C** The expression of indicated metastasis-inhibiting mRNAs was detected by qRT-PCR in MCPIP3-overexpressing A549 cells and NCI-H23 cells. Data are presented as mean ± SD; *$p < 0.05$, **$p < 0.01$, in unpaired t-test.

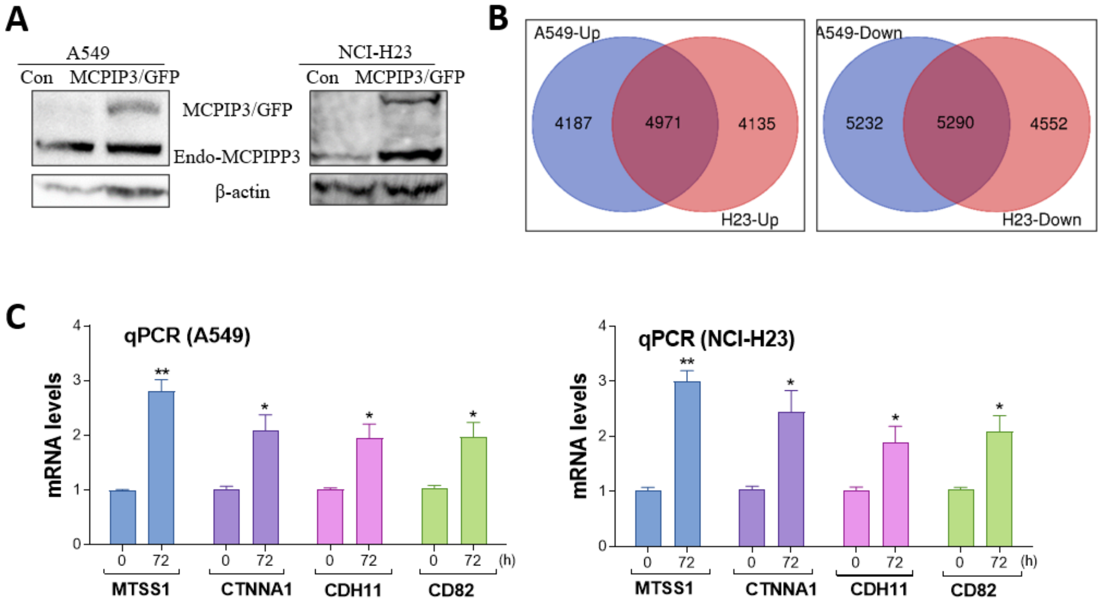

Figure S3. MCPIP3 selectively downregulated the expression of the metastasis-promoting gene METAP2 by destabilizing its mRNA via the RNase domain. A RNA-IP followed by RT-PCR detection showing that the indicated metastasis-inhibiting mRNAs were not pulled down by MCPIP3/GFP fusion protein. B Schematic representation of the luciferase reporters containing the 3'UTRs of metastasis-promoting genes. **C** The 3'UTR sequences from different species for the indicated metastasis-promoting genes were aligned using DNAMAN software, and the stem–loop sequences were predicted by RNAfold web server. **D** Half-lives of PNN and CTBP1 mRNAs were measured in MCPIP3-overexpressing A549 cells.

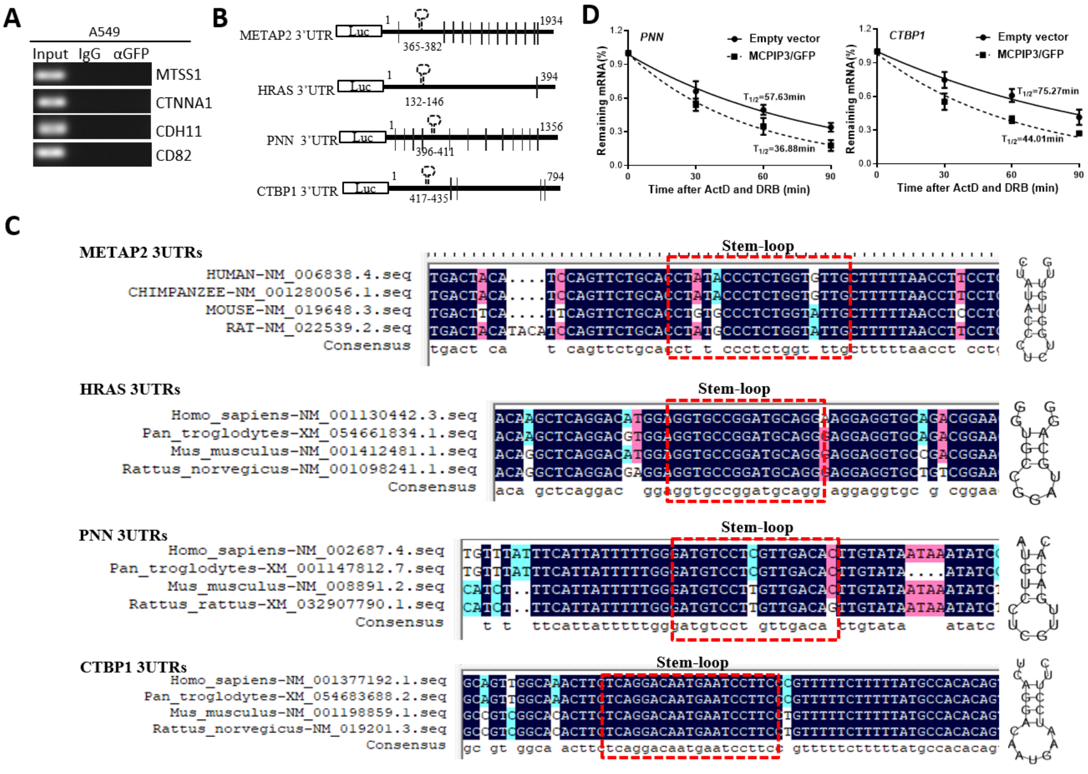

Figure S4. MCPIP2 depletion increased proangiogenic mRNAs stability and promoted tumor angiogenesis. A Half-life of PNN mRNA was measured in MCPIP3-knocking down A549 cells. **B** Representative images of cells migration from A549 cells with MCPIP3-knocking down alone or co-knockdown of MCPIP3 and METAP2 in the wound-healing assay.

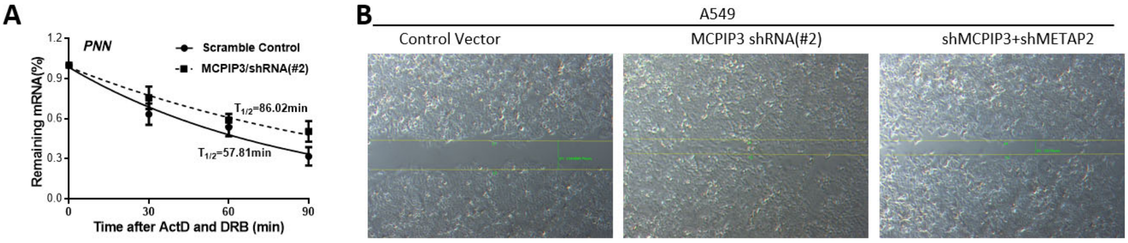

Figure S5. IGF2BP3 interacts with the common stem–loop structure to stabilize METAP2 mRNA and promote lung cancer metastasis. A RNA-seq data showing the expression of the indicated metastasis-promoting genes after IGF2BP3 overexpression in NCI-H23 cells. **B** The mRNA expression of METAP2 was measured by qRT-PCR after MCPIP3 or IGF2BP3 overexpression alone, or co-overexpression of the two RBPs in A549 cells. Data are presented as mean ± SD; *$p < 0.05$, in unpaired *t*-test.

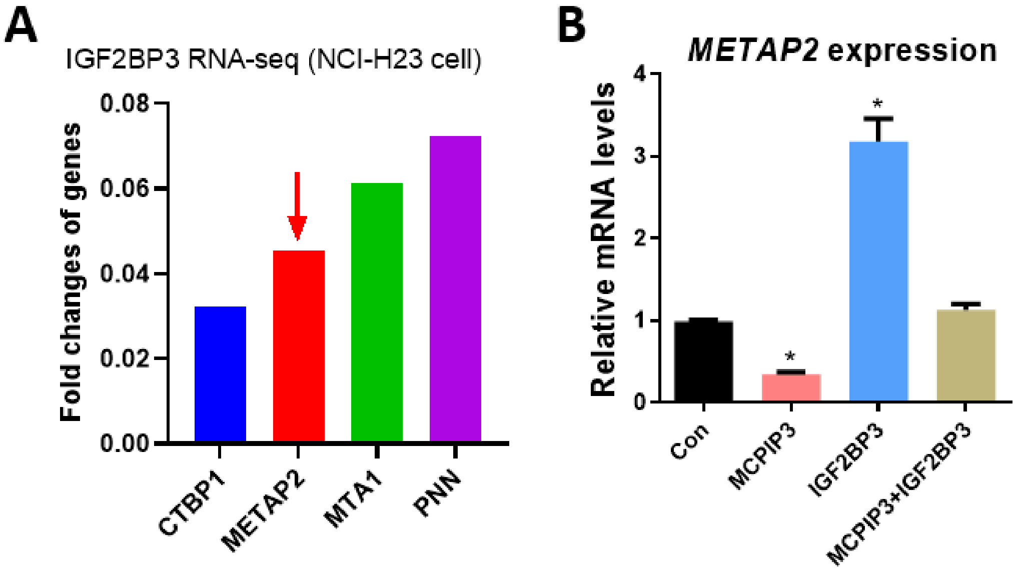

Figure S6. IGF2BP3 and MCPIP3 potentially antagonize metastasis-promoting genes expression and lung cancer cell metastasis. A The mRNA expression of *IGF2BP3* in human LUSC and normal tissues was analyzed using UALCAN online tool. **B** IGF2BP3 protein expression in human LUS and normal tissues was displayed by the CPTAC database. **C** Tumor volume of IGF2BP3-overexpressing A549 tumors was recorded after the mice sacrificed. **D** Pearson's correlation analysis between *MCPIP3* (up) *or IGF2BP3* (down) and the indicated metastasis-promoting genes expression in human LUAD patients were performed with R2 (http://r2.amc.nl). Data are presented as mean ± SD; ****p* < 0.001, in unpaired ***t***-test.

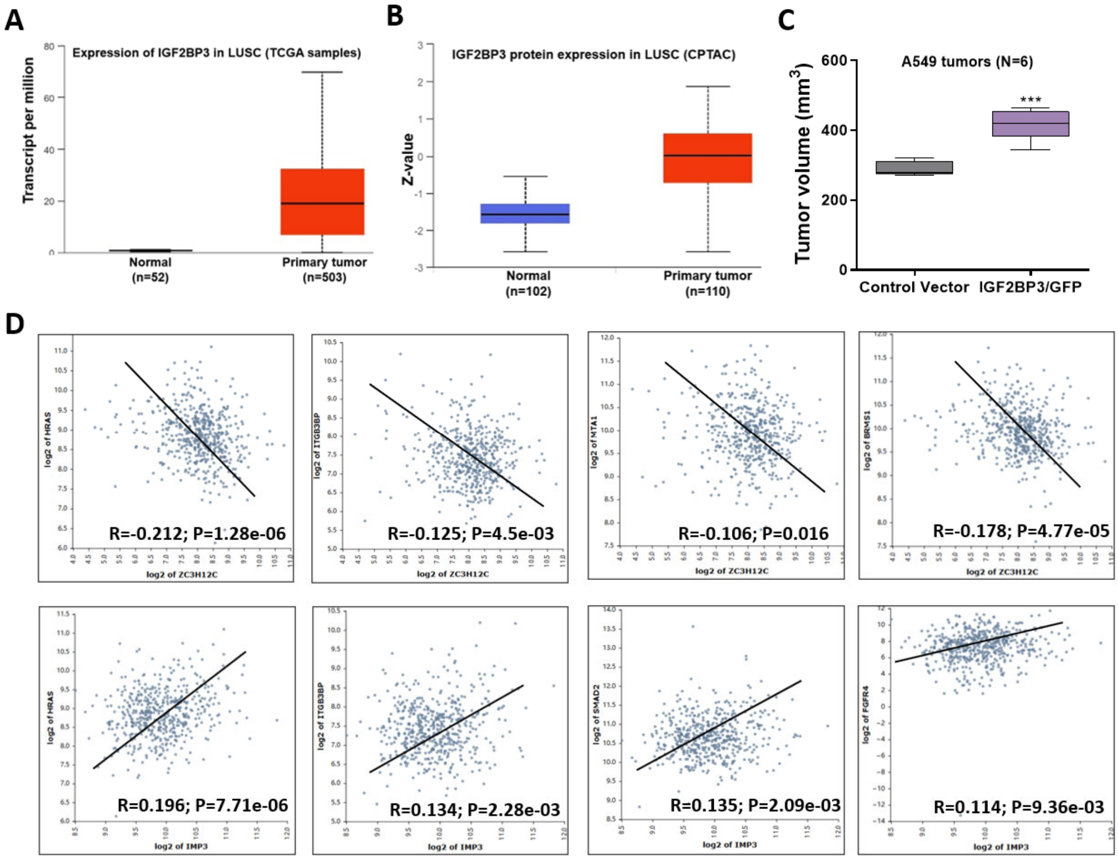

Supplement: Supplementary file 1 [file cancers-18-02384-s001.zip › cancers-4393166-supplementary figures.pdf]
