# Supplementary material for: RNA-Binding Proteins MCPIP3 and IGF2BP3 Antagonize Lung Cancer Metastasis by Reversibly Regulating METAP2 Expression via mRNA Stability
Source: Cancers (Basel). 2026 Jul 24;18(15):2384. doi: 10.3390/cancers18152384 (PMC13465609; doi:10.3390/cancers18152384)
Supplement: Supplementary file 1 [file cancers-18-02384-s001.zip › Table S1. PCR Primers and RNA-EMSA Probes Sequence.pdf]

Table S1. PCR Primers and RNA-EMSA Probes Sequence

Gene name	Primer (sense) (5'-3')	Primer (anti-sense) (5'-3')
HuMCPIP3 (qPCR)	ATA TGG AAA GAA GTG TAC CTA TGG ACA	TAG GTC TTG GTA GAC TTG TGT CCT TAT
HuGAPDH (qPCR)	GCTTGTCATCAATGGAAATCCCATCAC	TGATCTTGAGGCTGTTGTCATACTTCTC
HuMETAP2 (qPCR)	AGATGAAGATGATGAAGATGGAGATGGCG	CCAAATCTCTTCACTTGCCTGATCTAATGC
HuHRAS (qPCR)	ATA CGA CCC CAC TAT AGA GGA TTC CTA	GTT TGA TCT GCT CCC TGT ACT GGT
HuPNN (qPCR)	AAA GAG AGT CTT AAG AAC GTG GAT GAG	TGC TGG CTT TTT AAC ATC ATC ATC CT
HuCTBP1 (qPCR)	TTT TGA CAA CAT CGA CAT CAA GTC	CTA GAA GAG CAC GTT GAA GCC GAA
HuMTSS1 (qPCR)	GCAGAAGAAAGCAAAAAAAGGGAGAGGT	TGGTCAGGCTTTTTAGATCTTCCGAGAT
HuCTNNA1 (qPCR)	ACT TTT GGT ATT GAT TGA AGC TGC	GTT TAC TCT GTG GTT TTG CTG CTA A
HuCDH11 (qPCR)	TAG TGT ACA GTA TCC TCG AAG GAC	GTC TGG ATC TTT AGC TTT CAC TCT T
HuCD82 (qPCR)	AGC CTG TAT CAA AGT CAC CAA ATA C	GAA GCC CAT GAG CAT AGT GAC TG
HuMETAP2 (full-length 3'UTR)	TTCTAGAAC TTA GTC CAA AGC CAC CTC AAC ACC TTT A	GGCCGGCCGCA CTA TTG TTA AAC ATC AAA CTA ATA A
HuHRAS (full-length 3'UTR)	TTCTAGACG CAG GTG AGG GGG ACT CCC AGG GCG	GGCCGGCCACC ATC CAA TAA TTT ACT GTG ATC CCA TC
HuPNN (full-length 3'UTR)	TTCTAGATGG AAG AAG CCA GGC TTT CTT AGC CAT TCT	GGCCGGCCTAT GCA TTT ACT GTT TTA ATA ATT ACT CCA A
HuCTBP1 (full-length 3'UTR)	TTCTAGACC CGG GAG GAG CTC TCC AGC CTC	GGCCGGCCTGG CTC TTT CCA GGA TTT TTA TTT CTT TAA AAT A
HuMETAP2 (3'UTR-mut1)	AGT TCT GCA CCT ACC CCC TCT GGAATT GCT TTT TA	GGATGTAGTCATTCAAAGCATTCAAGTAAG AATATGGCA
HuMETAP2 (3'UTR-mut2)	AGT TCT GCA CCT AGT CCC TCT GGACTT GCT TTT TAA	GGATGTAGTCATTCAAAGCATTCAAGTAAG AATATGGCA
HuMETAP2 (RNA-ChIP)	GTT AGG AAT GAC TTA TAC GTT TTG TTT T	AGC TAT TTA TCC TGT CCA GAA TTA TAA C
METAP2 (WT-Probe):	5'- CUAUACCCUCUGGUGUUG -3'	
METAP2 (mut-Probe):	5'- CUACCCCCUCUGGAAUUG -3'	
