# Supplementary material for: RNA-Binding Proteins MCPIP3 and IGF2BP3 Antagonize Lung Cancer Metastasis by Reversibly Regulating METAP2 Expression via mRNA Stability
Source: Cancers (Basel). 2026 Jul 24;18(15):2384. doi: 10.3390/cancers18152384 (PMC13465609; doi:10.3390/cancers18152384)
Supplement: Supplementary file 1 [file cancers-18-02384-s001.zip › Table S2. PCRarray analysis of human metastasis-related genes regulated by MCPIP3.pdf]

Table S2. PCRarray analysis of human metastasis-related genes regulated by MCPIP3

Gene Symbol	Gene Name	Control	MCPIP3-Overexpression	fold change
APC	Adenomatous polyposis coli	**29.82**	**29.86**	0.16
BRMS1	Breast cancer metastasis suppressor 1	**29.86**	**29.81**	0.35
CCL7	Chemokine (C-C motif) ligand 7	-	-	NA
CD44	CD44 molecule (Indian blood group)	**22.42**	**23.28**	-0.27
CD82	CD82 molecule	**24.54**	**24.29**	0.42
CDH1	Cadherin 1, type 1, E-cadherin (epithelial)	**31.28**	**30.97**	0.47
CDH11	Cadherin 11, type 2, OB-cadherin (osteoblast)	-	-	NA
CDH6	Cadherin 6, type 2, K-cadherin (fetal kidney)	-	-	NA
CDKN2A	Cyclin-dependent kinase inhibitor 2A (melanoma, p16, inhibits CDK4)	**28.69**	**29.22**	-0.08
CDH4	Chromodomain helicase DNA binding protein 4	**36.65**	**36.33**	0.66
COL4A2	Collagen, type IV, alpha 2	**30.25**	**29.99**	0.59
CST7	Cystatin F (leukocystatin)	**35.64**	**35.38**	0.41
CTBP1	C-terminal binding protein 1	**30.32**	**30.96**	-0.15
CTNNA1	Catenin (cadherin-associated protein), alpha 1, 102kDa	**24.67**	**24.86**	0.16
CTSK	Cathepsin K	**29.45**	**29.71**	0.10
CTSL1	Cathepsin L1	**24.32**	**24.43**	0.23
CXCL12	Chemokine (C-X-C motif) ligand 12	-	-	NA
CXCR2	Chemokine (C-X-C motif) receptor 2	-	-	NA
CXCR4	Chemokine (C-X-C motif) receptor 4	**34.32**	**35.97**	-0.58
DENR	Density-regulated protein	**26.67**	**26.92**	0.12
EPHB2	EPH receptor B2	**27.36**	**27.38**	0.31
ETV4	Ets variant 4	**28.16**	**28.25**	0.25
EWSR1	Ewing sarcoma breakpoint region 1	**25.22**	**25.12**	0.42
FAT1	FAT tumor suppressor homolog 1 (Drosophila)	**27.28**	**27.97**	-0.18
FGFR4	Fibroblast growth factor receptor 4	**28.57**	**29.62**	-0.36
FLT4	Fms-related tyrosine kinase 4	-	-	NA
FN1	Fibronectin 1	**30.86**	**31.76**	-0.29
FXYD5	FXYD domain containing ion transport regulator 5	**25.43**	**25.36**	0.39
GNRH1	Gonadotropin-releasing hormone 1 (luteinizing-releasing hormone)	**27.18**	**28.13**	-0.32
HGF	Hepatocyte growth factor	**32.46**	**34.76**	-0.73
HPSE	Heparanase	**29.78**	**29.81**	0.30
HRAS	V-Ha-ras Harvey rat sarcoma viral oncogene homolog	**27.89**	**27.91**	0.31
HTATIP2	HIV-1 Tat interactive protein 2, 30kDa	**34.30**	**34.46**	0.19

IGF1	Insulin-like growth factor 1 (somatomedin C)	-	-	NA
IL18	Interleukin 18	**28.78**	**31.21**	-0.75
IL1B	Interleukin 1, beta	**39.46**	**39.98**	-0.07
ITGA7	Integrin, alpha 7	**30.26**	**30.01**	0.58
ITGB3	Integrin, beta 3	**29.87**	**31.23**	-0.49
KISS1	KiSS-1 metastasis-suppressor	**35.36**	**36.48**	-0.39
KISS1R	KISS1 receptor	-	-	NA
KRAS	V-Ki-ras2 Kirsten rat sarcoma viral oncogene homolog	**26.61**	**30.83**	-0.93
MCAM	Melanoma cell adhesion molecule	**28.67**	**29.21**	-0.09
MDM2	Mdm2 p53 binding protein homolog (mouse)	**26.36**	**27.13**	-0.22
MET	Met proto-oncogene (hepatocyte growth factor receptor)	**27.27**	**30.22**	-0.83
METAP2	Methionyl aminopeptidase 2	**27.56**	**29.69**	-0.70
MGAT5	Mannosyl (alpha-1,6-)-glycoprotein beta-1,6-N-acetyl-glucosaminyltransferase	**31.19**	**31.24**	0.28
MMP10	Matrix metallopeptidase 10 (stromelysin 2)	-	-	NA
MMP11	Matrix metallopeptidase 11 (stromelysin 3)	**37.91**	**37.82**	0.41
MMP13	Matrix metallopeptidase 13 (collagenase 3)	-	-	
MMP2	Matrix metallopeptidase 2	**36.56**	**36.22**	0.68
MMP3	Matrix metallopeptidase 3 (stromelysin 1, progelatinase)	-	-	NA
MMP7	Matrix metallopeptidase 7 (matrilysin, uterine)	**32.23**	**34.18**	-0.66
MMP9	Matrix metallopeptidase 9 (gelatinase B, 92kDa gelatinase, 92kDa type IV collagenase)	-	-	NA
MTA1	Metastasis associated 1	**31.12**	**31.98**	-0.27
MTSS1	Metastasis suppressor 1	**29.19**	**28.63**	0.96
MYC	V-myc myelocytomatosis viral oncogene homolog (avian)	**27.27**	**27.96**	-0.18
MYCL1	V-myc myelocytomatosis viral oncogene homolog 1, lung carcinoma derived (avian)	**25.74**	**25.28**	0.38
NF2	Neurofibromin 2 (merlin)	**26.76**	**26.79**	0.30
NME1	Non-metastatic cells 1, protein (NM23A) expressed in	**31.12**	**31.11**	0.34
NME4	Non-metastatic cells 4, protein expressed in	**31.33**	**31.29**	0.36
NR4A3	Nuclear receptor subfamily 4, group A, member 3	**29.59**	**30.13**	-0.09
PLAUR	Plasminogen activator, urokinase receptor	**29.83**	**29.92**	0.25
PNN	Pinin, desmosome associated protein	**27.65**	**27.69**	0.29

PTEN	Phosphatase and tensin homolog	28.68	28.29	0.74
RB1	Retinoblastoma 1	33.43	33.01	0.78
RORB	RAR-related orphan receptor B	37.35	37.97	-0.14
RPSA	Ribosomal protein SA	30.28	31.51	-0.43
SERPINE1	Serpin peptidase inhibitor, clade E (nexin, plasminogen activator inhibitor type 1), member 1	34.59	34.13	0.83
SET	SET nuclear oncogene	27.32	28.39	-0.37
SMAD2	SMAD family member 2	28.84	29.73	-0.27
SMAD4	SMAD family member 4	26.57	26.48	0.35
SRC	V-src sarcoma (Schmidt-Ruppin A-2) viral oncogene homolog (avian)	29.45	30.22	-0.22
SSTR2	Somatostatin receptor 2	25.55	25.34	0.54
SYK	Spleen tyrosine kinase	29.92	30.84	-0.30
TCF20	Transcription factor 20 (AR1)	31.25	31.26	0.32
TGFB1	Transforming growth factor, beta 1	28.33	28.11	0.55
TIMP2	TIMP metallopeptidase inhibitor 2	27.33	26.82	0.89
TIMP3	TIMP metallopeptidase inhibitor 3	29.39	29.11	0.61
TIMP4	TIMP metallopeptidase inhibitor 4	38.84	38.66	0.51
TNFSF10	Tumor necrosis factor (ligand) superfamily, member 10	31.29	31.22	0.39
TP53	Tumor protein p53	27.87	27.43	0.12
TRPM1	Transient receptor potential cation channel, subfamily M, member 1	38.54	38.88	0.05
TSHR	Thyroid stimulating hormone receptor	39.42	39.70	0.09
VEGFA	Vascular endothelial growth factor A	27.36	28.89	-0.54
ACTB	Actin, beta	21.22	22.41	
B2M	Beta-2-microglobulin	24.12	23.35	
GAPDH	Glyceraldehyde-3-phosphate dehydrogenase	**19.21**	**19.34**	
HPRT1	Hypoxanthine phosphoribosyltransferase 1	28.24	28.73	
RPLP0	Ribosomal protein, large, P0	18.58	18.44	
HGDC	Human Genomic DNA Contamination			
RTC	Reverse Transcription Control			
RTC	Reverse Transcription Control			
RTC	Reverse Transcription Control			
PPC	Positive PCR Control	21.33	21.42	
PPC	Positive PCR Control	21.36	21.69	
PPC	Positive PCR Control	21.41	21.88	
